# Supplementary material for: Biochemical monitoring after initiation of aldosterone antagonist therapy in users of renin–angiotensin system blockers: a UK primary care cohort study
Source: BMJ Open. 2017 Nov 16;7(11):e018153. doi: 10.1136/bmjopen-2017-018153 (PMC5701996; doi:10.1136/bmjopen-2017-018153)
Supplement: Supplementary data [file bmjopen-2017-018153supp001.pdf]

Supplementary Information

Table S1: Summary of clinical guideline recommendations for blood testing after initiation of AA

	NICE HF*	NICE HF Practical Guidelines Appendix D	NICE HF Practical Guidelines Appendix M	NICE Hypertension§	British National Formulary	American Guidelines∞	European Guidelines
≤7 days	*na*	x	x	*na*	x	x	x
≤1 month	*na*	x	x	x	X	x	x
≤2 month	*na*	x	x	*na*	X	x	x
≤3 month	*na*	x	x	*na*	X	x	x
≤6 month	*na*	x	x	*na*	X	x	x
≤9 month	*na*	*na*	x	*na*	X	x	x
≤12 month	*na*	x	x	*na*	X	x	x
How frequently after 1st year	*na*	6 monthly	6 monthly	*na*	6 monthly	3 monthly	4 monthly
Discontinue if K≥6mmol/L	*na*	x	x	*na*	*na*	x∞∞	x
Discontinue if creatinine ≥220µmol/L	*na*	x	x†	*na*	*na*	*na***	x‡
Discontinue if relative changes between baseline creatinine and follow up creatinine or eGFR ≥X%	*na*	*na*	*na*	*na*	*na*	*na***	*na*

* NICE HF guidelines recommend monitoring within two weeks after a medication change, but refer to Appendix D for guidance on monitoring for hyperkalaemia and renal function deterioration

† Recommends discontinuing if creatinine ≥200µmol/L

§ Recommends blood testing as required after testing within one month of AA initiation/increased diuretic use

∞ Recommends blood testing within 2-3 days of AA initiation, and again at 7 days.

∞∞ Recommends discontinuation at potassium > 5.5mEq/L

** Does not provide strict stopping/starting criteria according to serum creatinine thresholds. Suggests that risk of hyperkalaemia increases with worsening renal function and in elderly patients, or patients with low muscle mass in whom serum creatinine does not accurately reflect GFR, determination that GFR is >30ml/min/1.73m^2 is recommended.

‡ Recommends discontinuing if creatinine ≥310µmol/L or eGFR <20ml/min/1.73m^2
European Guidelines: “2016 ESC Guidelines for the diagnosis and treatment of acute and chronic heart failure”

American Guidelines: “2013 ACCF/AHA Guideline for the Management of Heart Failure”
NICE: National Institute for Health and Care Excellence
HF: Heart Failure
GP: General Practitioner
“x” refers to when the row condition is satisfied, “na” refers to not mentioned or not satisfied.

SI Table 2: Proportion of non-hospitalised patients taking ACEI/ARB who initiate aldosterone antagonists in UK primary care 2004-2014 with blood tests with 14 days of AA initiation

Non-hospitalised population	Monitoring ≤14 days post initiation (n., %)	No monitoring ≤14 days post initiation (n., %)
n=5787	1892 (32.7)	3897 (67.3)

Proportions for monitoring use 5787 as denominator. Non-hospitalisation refers to not having a hospitalisation in the 30 days prior to or post initiation of AA.

SI Table 3: Association between non-hospitalised patient characteristics and follow up monitoring within two weeks of initiation of an aldosterone antagonist

	OR (95% CI)	
	**Age and sex adjusted**	**Fully adjusted**
**Male**	*ref*	*ref*
**Female**	0.88 (0.78 – 0.99)	0.91 (0.80 – 1.03)
**Age (years)**		
<50	0.38 (0.28 – 0.53)	0.42 (0.30 – 0.58)
50-59	0.69 (0.56 – 0.85)	0.75 (0.60 – 0.93)
60-64	0.83 (0.66 – 1.06)	0.89 (0.70 – 1.13)
65-69	0.93 (0.76 – 1.14)	0.95 (0.77 – 1.17)
70-75	*ref*	*ref*
76-79	0.91 (0.75 – 1.11)	0.92 (0.75 – 1.12)
80+	1.13 (0.95 – 1.34)	1.11 (0.92 – 1.32)
**eGFR category (ml/min/1.73m^2)**		
≥60	*ref*	*ref*
45-59	1.00 (0.86 – 1.16)	1.02 (0.87 – 1.19)
30-44	1.10 (0.93 – 1.31)	1.10 (0.92 – 1.32)
15-29	1.13 (0.82 – 1.57)	1.15 (0.83 – 1.60)
**Comorbidities**		
Hypertension	0.98 (0.86 – 1.12)	0.96 (0.83 – 1.60)
Ischaemic Heart Disease	1.09 (0.97 – 1.23)	1.04 (0.93 – 1.17)
Heart Failure	1.17 (1.04 – 1.32)	1.11 (0.97 – 1.26)
Arrhythmia	1.16 (1.03 – 1.31)	1.10 (0.97 – 1.25)
Diabetes	1.16 (1.02 – 1.33)	1.17 (1.02 – 1.34)
Peripheral Arterial Disease	1.13 (0.93 – 1.38)	1.06 (0.87 – 1.31)
**Calendar time**		
2004-2006	*ref*	*ref*
2007-2009	1.26 (1.07 – 1.49)	1.25 (1.06 – 1.49)
2010-2014	1.36 (1.15 – 1.61)	1.35 (1.13 – 1.60)
**Baseline potassium**		
<5.0mmol/L	*ref*	*ref*
5.0-5.5mmol/L	1.05 (0.87 – 1.27)	1.01 (0.84 – 1.23)
>5.5mmo/L	0.87 (0.59 – 1.30)	0.85 (0.57 – 1.26)

eGFR–estimated Glomerular Filtration Rate.
Fully adjusted: adjusted for age, gender, eGFR category, hypertension, heart failure, ischaemic heart disease, arrhythmias, diabetes, peripheral arterial disease, baseline potassium and calendar time. Further adjustment for lifestyle covariates and socioeconomic status made marginal difference to all results, thus these variables are not included in models shown.

SI Table 4: Proportion of non-hospitalised patients with adverse biochemical values on testing within 2 months of AA initiation and number subsequently discontinuing aldosterone antagonist

	**Hyperkalaemia (>=6mmol/L)‡**	**Creatinine (≥220µmol/L)**	**≥30% Change in Creatinine**
Number with adverse biochemical values (n,%)*	60/3698 (1.6%)	75/3757 (2.0%)	374/3757 (10.0%)
Number discontinuing AA (n, %)∞	30/60 (50.0%)	29/75 (38.7%)	109/374 (29.1%)

*serum potassium and creatinine values on first blood test within two months of AA initiation.
‡Missing data for 59 people for first follow-up potassium value.
§Discontinuation defined as no further prescriptions of AA after blood test plus 30 days.
AA–aldosterone antagonist.

SI Table 5: Associations between non-hospitalised patient characteristics and adverse biochemical values after aldosterone antagonist initiation

	OR (95% CI)		
	**Hyperkalaemia* (>=6mmol/L)**	**Creatinine* (≥220µmol/L)**	**≥30% Change in Creatinine**
**Male**	*ref*	*ref*	*ref*
**Female**	1.53 (0.93 – 2.51)	0.78 (0.50 – 1.23)	1.52 (1.22 – 1.90)
**Age (years)**			
<50	-	0.80 (0.22 – 2.87)	0.37 (0.16 – 0.85)
50-59	0.47 (0.15 – 1.45)	0.45 (0.16 – 1.30)	0.67 (0.40 – 1.13)
60-64	0.57 (0.18 – 1.79)	0.67 (0.23 – 1.94)	0.79 (0.47 – 1.30)
65-69	0.41 (0.13 – 1.28)	0.20 (0.05 – 0.81)	0.81 (0.52 – 1.26)
70-75	*ref*	*ref*	*ref*
76-79	0.93 (0.44 - 1.97)	1.34 (0.65 – 2.78)	1.05 (0.73 – 1.51)
80+	0.82 (0.41 - 1.64)	1.22 (0.63 – 2.37)	1.25 (0.87 – 1.80)
**eGFR category (ml/min/1.73m^2)**			
≥60	*ref*		
45-59	3.36 (1.40 – 8.05)	5.02 (1.24 – 20.24)	0.84 (0.64 – 1.10)
30-44	6.01 (2.42 – 14.89)	47.79 (13.70 – 166.76)	1.11 (0.81 – 1.53)
15-29	8.69 (2.51 – 30.10)	875.60 (240.46 – 3188.32)	1.23 (0.75 – 2.04)
**Comorbidities**			
Hypertension	1.11 (0.51 – 2.37)	1.12 (0.60 -2.08)	1.30 (0.98 - 1.73)
Ischaemic Heart Disease	0.52 (0.28 – 0.96)	0.59 (0.33 – 1.07)	1.03 (0.84 -1.27)
Heart Failure	0.72 (0.42 – 1.22)	0.98 (0.55 -1.73)	0.92 (0.73 - 1.15)
Arrhythmia	0.93 (0.54 – 1.61)	0.57 (032 – 1.01)	1.03 (0.82 -1.29)
Diabetes	0.96 (0.53 – 1.74)	1.10 (0.61 – 1.98)	0.97 (0.76 – 1.24)
Peripheral Arterial Disease	1.05 (0.38 – 2.87)	0.55 (0.21 – 1.40)	0.89 (0.59 - 1.34)
**Calendar time**			
2004-2006			
2007-2009	0.94 (0.42 – 2.07)	1.19 (0.60 – 2.38)	1.01 (0.75 – 1.35)
2010-2014	0.91 (0.46 – 1.77)	0.76 (0.40 – 1.47)	0.96 (0.72 – 1.27)
**Baseline potassium**			
<5.0mmol/L			
5.0-5.5mmol/L	2.52 (1.31 – 4.83)	*n/a*	*n/a*
>5.5mmo/L	5.26 (1.87 – 14.81)		

*Adverse serum potassium and creatinine values on first blood test within two months of AA initiation.
N = 3,757 except missing data for 59 people for first follow-up potassium value.
Only fully adjusted model is shown for clarity. Adjusted for age, gender, eGFR category, hypertension, heart failure, ischaemic heart disease, arrhythmias, diabetes, peripheral arterial disease, baseline potassium (hyperkalemia model only) and calendar time. Further adjustment for lifestyle covariates and socioeconomic status made marginal difference to all results and is not presented.
eGFR–estimated Glomerular Filtration Rate.
