# Supplementary material for: Biochemical monitoring after initiation of aldosterone antagonist therapy in users of renin–angiotensin system blockers: a UK primary care cohort study
Source: BMJ Open. 2017 Nov 16;7(11):e018153. doi: 10.1136/bmjopen-2017-018153 (PMC5701996; doi:10.1136/bmjopen-2017-018153)
Supplement: Author's manuscript [file bmjopen-2017-018153.draft_revisions.pdf]

BMJ Open is committed to open peer review. As part of this commitment we make the peer review history of every article we publish publicly available.

When an article is published we post the peer reviewers' comments and the authors' responses online. We also post the versions of the paper that were used during peer review. These are the versions that the peer review comments apply to.

The versions of the paper that follow are the versions that were submitted during the peer review process. They are not the versions of record or the final published versions. They should not be cited or distributed as the published version of this manuscript.

BMJ Open is an open access journal and the full, final, typeset and author-corrected version of record of the manuscript is available on our site with no access controls, subscription charges or pay-per-view fees (http://bmjopen.bmj.com).

If you have any questions on BMJ Open's open peer review process please email editorial.bmjopen@bmj.com

Biochemical monitoring in combined renin-angiotensin system blocker - aldosterone antagonist therapy

Journal:	*BMJ Open*
Manuscript ID	bmjopen-2017-018153
Article Type:	Research
Date Submitted by the Author:	14-Jun-2017
Complete List of Authors:	Sinnott, Sarah-Jo; London School of Hygiene and Tropical Medicine, Non-communicable disease epidemiology<br>Mansfield, Kathryn; London School of Hygiene and Tropical Medicine, Non-communicable disease epidemiology<br>Schmidt, Morten; London School of Hygiene and Tropical Medicine, Non-communicable disease epidemiology; Aarhus Universitet, Dept of Clinical Epidemiology<br>Bhaskaran, Krishnan; London School of Hygiene and Tropical Medicine, Non-communicable disease epidemiology<br>Smeeth, Liam; London School of Hygiene and Tropical Medicine, Non-communicable disease epidemiology<br>Nitsch, Dorothea; London School of Hygiene and Tropical Medicine, Non-communicable disease epidemiology<br>Tomlinson, Laurie; London School of Hygiene and Tropical Medicine, Non-communicable disease epidemiology
**Primary Subject Heading**:	Cardiovascular medicine
Secondary Subject Heading:	Cardiovascular medicine, Epidemiology, Health services research, Pharmacology and therapeutics
Keywords:	Heart failure < CARDIOLOGY, Hypertension < CARDIOLOGY, CLINICAL PHARMACOLOGY

SCHOLARONE™ Manuscripts

Biochemical monitoring in combined renin-angiotensin system blockade - aldosterone antagonist therapy

Authors:

Sarah-Jo Sinnott[1], Kathryn E Mansfield[1], Morten Schmidt[1,2] Krishnan Bhaskaran[1], Liam Smeeth[1], Dorothea Nitsch[1] Laurie A Tomlinson[1]

1. Department of non-communicable disease epidemiology, London School of Hygiene and Tropical Medicine, Keppel St, London. WC1E7HT
2. Department of Clinical Epidemiology, Aarhus University Hospital.

Corresponding Author:

Sarah-Jo Sinnott
Department of non-communicable disease epidemiology, London School of Hygiene & Tropical Medicine, Keppel St, London, WC1E7HT
Email: sarah-jo.sinnott@lshtm.ac.uk
Phone 02072994821

Word count

~3400

Abstract count

249

Tables and figures

1 Figure

5 Tables

Keywords

Hypertension, heart failure, electronic medical records, health care delivery, drug monitoring

For peer review only - http://bmjopen.bmj.com/site/about/guidelines.xhtml

COPYRIGHT/LICENCSE FOR PUBLICATION

"The Corresponding Author has the right to grant on behalf of all authors and does grant on behalf of all authors, an exclusive licence (or non exclusive for government employees) on a worldwide basis to the BMJ Publishing Group Ltd to permit this article (if accepted) to be published in BMJ editions and any other BMJPGL products and sublicences such use and exploit all subsidiary rights, as set out in our licence."

CONTRIBUTORSHIP

LAT had the idea for the study and acquired data permissions. SJS, MS, KEM, DN and LAT designed the study. SJS, KEM and MS managed the data and established the cohort. SJS did the analyses. All authors participated in the discussion and interpretation of the results. SJS organised the writing and wrote the initial drafts. All authors critically revised the manuscript for intellectual content and approved the final version. SJS is the guarantor.

TRANSPARENCY

SJS affirms that the manuscript is an honest, accurate, and transparent account of the study being reported; that no important aspects of the study have been omitted; and that any discrepancies from the study as planned have been explained.

ETHICS

The protocol for this study was approved by London School of Hygiene and Tropical Medicine Ethics Committee (No. 6536) and the Independent Scientific Advisory Committee (ISAC) for Medicines and Healthcare Products Regulatory Agency (No. 16_025A).

FUNDING

SJS is supported by a Wellcome Trust Sir Henry Wellcome Fellowship [107340/Z/15/Z]. LS is supported by a Wellcome Trust Senior Research Fellowship in Clinical Science [098504/Z/12/Z]. LAT and KEM are supported by a Wellcome Trust Intermediate Clinical Fellowship to LAT [101143/Z/13/Z]. KB is supported by a Sir Henry Dale Fellowship jointly funded by the Wellcome Trust and the Royal Society [107731/Z/15/Z]. MS was supported by the A.P. Møller Foundation for the Advancement of Medical Science, Snedkermester Sophus Jacobsen & Hustru Astrid Jacobsens Fond, and Christian og Ottilia Brorsons Rejselegat for yngre videnskabsmænd og–kvinder.

COMEPETING INTERESTS

All authors have completed the ICMJE uniform disclosure form at www.icmje.org/coi_disclosure.pdf and declare: SJS, KEM, MS, DN and LAT nothing to declare; LS reports grants from Wellcome Trust and British Heart Foundation during the conduct of the study, grants from Wellcome Trust, Medical Research Council, National Institute for Health Research and the European Union outside the submitted work, personal fees from GSK for advisory work unrelated to the submitted work, grant funding from GSK for academic research unrelated to the submitted work, acts as an unpaid steering committee chair for AstraZeneca for a randomised trial unrelated to the submitted work, and is a trustee of the British Heart Foundation. KB declares grants from the Wellcome Trust, Royal Society, MRC, NIHR and BHF outside the submitted work.

For peer review only

For peer review only - http://bmjopen.bmj.com/site/about/guidelines.xhtml

ABSTRACT

Objective

To determine the frequency of biochemical monitoring after initiation of aldosterone atagonists, in patients also using angiotensin-converting enzyme inhibitor/angiotensin receptor blockers (ACEI/ARB).

Setting

UK primary care

Participants

ACEI/ARB users who initiated an AA between 2004-2014.

Outcomes

We calculated the proportions with: 1) biochemical testing ≤2 weeks post AA initiation; 2) adverse biochemical values ≤2 months (potassium ≥6mmol/L; creatinine ≥220µmol/L; ≥30% increase in creatinine from baseline); 3) discontinuers of AA in those with an adverse biochemical value. We used logistic regression to study patient characteristics associated with testing and adverse biochemical values.

Results

In 10,546 initiators of AA, 3,291 (31.2%) had a record of testing ≤2 weeks post initiation. Women and those aged <60 years compared to 70-74 years had lower odds of receiving testing. A total of 2.0% and 2.7% of those with follow-up testing ≤2months experienced potassium ≥6mmol/L and creatinine ≥220µmol/L respectively, while 13.5% had a ≥30% increase in creatinine. Baseline potassium (OR 3.59, 95% CI 2.43-5.32 for 5.0-5.5mmol/L compared to <5.0mmol/L) and eGFR 45-59mls/min/1.73m^2 (OR 2.06, 95% CI 1.26–3.35 compared to ≥60mls/min/1.73m^2) were independently predictive of potassium ≥6mmol/L. Women and those with diabetes had higher odds of ≥30% increase in creatinine.

Conclusion

Less than one-third of patients taking ACEI/ARB had testing within 2 weeks of initiating AA. Importantly, women had lower odds of testing but higher odds of deterioration in renal function.

For peer review only - http://bmjopen.bmj.com/site/about/guidelines.xhtml

STRENGTHS AND LIMITATIONS OF THE STUDY

- This is a population cohort study, based on electronic health records from UK primary care, examining whether users of renin-agiotensin system blockade and aldosterone antagonists (AA) in combination experience biochemical monitoring after initiation of AA.
- The population was not restricted by indication for therapy.
- Those who were hospitalised prior to or immediately after initiating an AA may have had missing data for test results in primary care data. In a sensitivity analysis, we used primary care data linked to hospital data to assess monitoring in a population that was not hospitalised. We found similar rates of monitoring and adverse events compared to the main analysis.

For peer review only

INTRODUCTION

Angiotensin-converting enzyme inhibitors or angiotensin receptor blockers (ACEI/ARB) and aldosterone antagonists (AA), such as spironolactone and eplenerone, are frequently used in combination. They provide reductions in morbidity and mortality for patients with heart failure [1] and reductions in blood pressure for patients with resistant hypertension.[2] However, users of these drugs are at risk of acute kidney injury [3], hyperkalaemia, and hyperkalaemia-associated mortality.[4]

The occurrence of adverse events associated with combined ACEI/ARB and AA use was recently highlighted in the UK with the publication of a drug safety notice from the Medicines and Healthcare products Regulatory Agency (MHRA). It reported on the increasing incidence of life-threatening hyperkalaemic adverse events in patients prescribed ACEI/ARB and spironolactone.[5]

To help avoid adverse events after initiation of AA, biochemical parameters should be monitored. At present, the National Institute of Health and Care Excellence (NICE) practical guidelines for heart failure recommend testing for potassium, creatinine, and estimated Glomerular Filtration Rate (eGFR) after one week, and at one, two, three, and six months, and six monthly thereafter, following initiation of an AA in heart failure.[6] These guidelines recommend stopping the AA if potassium ≥6mmol/L and if creatinine ≥220µmol/L.[6] NICE guidelines for hypertension state that testing for sodium, potassium and renal function should occur within one month after inititiation of AA, and as required thereafter.[7]

It is not known how well these guidelines are adhered to in the UK. Previous evidence on blood testing during AA treatment in the UK is historic, restricted to one geographical region, and did not specifically assess adherence to guideline recommended blood testing.[8] Data from North America suggest that recommended blood testing occurs in less than 50% of patients.[3, 9, 10] Poor monitoring of patients taking these drug combinations, as well as increasing use among patients at high risk of adverse outcomes, may help to explain the increased occurrence of hyperkalaemic events as reported by the MHRA.

Therefore, among a large, recent cohort of users of ACEI/ARB who initiated an AA, we sought to examine patterns of blood testing, and the occurrence of hyperkalaemia and renal impairment. Our aims were to determine: 1) the proportion of people initiating an AA who had testing with two weeks of initiation; 2) the patient characteristics associated with testing 3) the proportion of people who had adverse biochemical values after initiation of AA, and the proportion that then discontinued the AA and 4) the patient characteristics associated with adverse biochemical values.

For peer review only - http://bmjopen.bmj.com/site/about/guidelines.xhtml

For peer review only

METHODS

Data

The Clinical Practice Research Database (CPRD-GOLD) is a nationally representative repository of de-identified electronic medical records from primary care in the UK. It holds data on demographics, health related behaviours, test results, diagnoses, referrals and prescriptions for 4.4 million people currently alive with research-quality data.[11] It is one of the largest databases of longitudinal medical records from primary care globally and has been extensively validated.[11, 12] For this study, data on test results were extracted from these primary care records.

For this study, we used CPRD-GOLD data linked to Hospital Episodes Statistics (HES). This linkage is possible for 60% of English practices contributing to the CPRD-GOLD database. The HES database provides data on the primary diagnosis for a hospital admission, as well as other diagnoses and procedures carried out during that admission. The linkage thus provides a more complete picture of comorbidities, improves the accuracy of timing and in this study allowed the conduct of sensitivity analyses in those without hospital admissions.[13]

Population

We identified a cohort of HES-linked CPRD patients aged ≥18 years, who initiated ACEI/ARB treatment between April 1, 1997 and March 31, 2014.[14] We identified continuous courses of ACEI/ARB therapy by allowing for a 90-day gap between the end date of one prescription and the start of the next consecutive prescription (to allow for stock piling and medications prescribed in secondary care). Among this cohort, we identified people who subsequently became new users of AA in the period 2004-2014. New use of AA was defined as no use of AA in the year prior to first prescription. We then restricted the population to those with a record for creatinine monitoring in the year prior to AA initiation. We assumed that those with creatinine test results prior to and after AA initiation were also tested for potassium. If values for potassium were not present they were treated as missing. This approach avoided exclusion of patients whose blood sample may have been haemolysed, resulting in potassium value not reported. Patients with READ codes for end-stage renal disease and estimated glomerular filtration rates (eGFR) values corresponding with chronic kidney disease stage 5 prior to cohort entry were excluded. Patients were eligible for follow up until the earliest of; death, transfer out of practice, last data collection or end of the study (March 2014).

Covariates

We obtained information for all patients on age, gender, calendar time of AA initiation (2004-2006, 2007-2009, 2010-2014), and lifestyle factors. The closest records to AA initiation date were used for

For peer review only - http://bmjopen.bmj.com/site/about/guidelines.xhtml

determining smoking, alcohol and body mass index status using existing algorithms.[15] Ethnicity was categorised as white, black, south Asian, and other/mixed. We extracted data on the cardiovascular comorbidities and diabetes using data from both CPRD and HES. We calculated baseline eGFR using the most recent creatinine value from CPRD data prior to AA initiation and the Chronic Kidney Disease Epidemiology Collaboration (CKD-EPI) equation.[16] CPRD prescribing data were used to extract data on baseline medication use. Baseline potassium values were categorised as: <5mmol/L, 5–5.5mmol/L and >5.5mmol/L.

Outcomes and Statistical Analyses

Several guidelines recommend time periods for blood testing after initiation of an AA **(Supplementary Table 1)**. We assessed whether monitoring occurs in line with current NICE recommendations, with some modification. NICE practical guidelines for heart failure recommend blood testing within one week after AA initiation.[6] However, we calculated the proportion of AA initiators who had blood testing within two weeks of AA initiation to accommodate the practical challenges of immediate follow-up testing faced in clinical practice. In additional analyses, we also calculated the proportion of people who had testing 1) within seven days post AA initiation, 2) within one month, two months, 3 months, 6 months and 12 months post AA initiation and 3) on all recommended monitoring occasions.[3]

We used thresholds set out by the NICE practical guidelines for heart failure to calculate the proportion of patients who had an adverse biochemical value on their first blood test within two months of initiation.[6] Hyperkalaemia was a potassium of ≥6mmol/L and an adverse creatinine value indicating renal dysfunction was defined as creatinine ≥220μmol/L. We chose two months to accord with monitoring periods in previous clinical trials[17], and also because at the outset we expected that a majority of patients would have testing within this timeframe. We then calculated the proportion of those with adverse biochemical values who discontinued the AA. We used a conservative definition of discontinuation, to prevent misclassification of people who had blood tests at the beginning of a median length prescription (28 days). Therefore, we classified discontinuation as no further prescription of an AA beyond 30 days after the first post-initiation blood test, i.e., when the end date of the course of AA therapy occurred before the first blood test date plus 30 days.

Current practical guidelines for initiation of AA refer to an absolute level of creatinine to indicate renal dysfunction of concern and when the AA should be stopped.[6] However, this value reflects substantially different levels of eGFR depending on the age, gender and ethnicity of the patient. A proportional change in creatinine or eGFR is recommended by NICE guidelines to indicate significant

For peer review only - http://bmjopen.bmj.com/site/about/guidelines.xhtml

change in renal function for patients who initiate ACEI/ARB.[18] This measure is therefore clinically familiar and more closely indicates changes in renal function that may be associated with a new drug. Therefore, we also calculated the number of people who experienced a 30% relative increase in creatinine from baseline as an adverse biochemical finding. We used the most recent values for creatinine within one year prior to AA initiation and creatinine values on the first blood test within two months post AA initiation to calculate the relative change.

To assess patient level characteristics associated with testing within two weeks (versus not having testing within two weeks) we used logistic regression with robust standard errors to adjust for correlations between patients within practices.[9] The crude model adjusted for age and gender only, while the fully adjusted model adjusted for age, gender, eGFR category, cardiovascular comorbidities, diabetes, baseline potassium and calendar time. We did not include ethnicity in fully adjusted models due to missing data for approximately 50% of the population.[19] We also used logistic regression to assess patient characteristics associated with an adverse biochemical value (versus not having an adverse biochemical value), with adjustments for the same variables as in the prior model.

In a sensitivity analysis, we assessed the proportion of patients receiving blood tests among patients who were not hospitalised in the 30-day period prior to or after AA initiation. Data on laboratory tests are not available in the HES database, and test results may not always be sent from the hospital to the GP practice. Therefore, this analysis excluded patients who had blood tests in hospital, which would contribute to an apparently low observed level of testing in primary care.

We used Stata version 14 for all analyses.[20]

The protocol for this study was approved by London School of Hygiene and Tropical Medicine Ethics Committee (No. 6536) and the Independent Scientific Advisory Committee (ISAC) for Medicines and Healthcare Products Regulatory Agency (No. 16_025A).

For peer review only - http://bmjopen.bmj.com/site/about/guidelines.xhtml

RESULTS

From 466,271 continuous users of ACEI/ARB between 1997 and March 2014, 12,291 people initiated an AA between 2004-2014 and were eligible for inclusion. After further exclusions, 10,546 were ultimately included in the cohort. **(Figure 1)**. The population was 41% female and had a mean age of 71.8 years (SD 12.9) **(Table 1)**. Mean serum potassium was 4.4 mmol/L (SD 0.7). Approximately one fifth of the population had eGFR <30mls/min/1.73m^2 **(Table 1).** Spironolactone was the drug commenced for 9,917/10,546 (94%) of those initiating an AA, with the remainder initiating eplenerone.

For peer review only

For peer review only - http://bmjopen.bmj.com/site/about/guidelines.xhtml

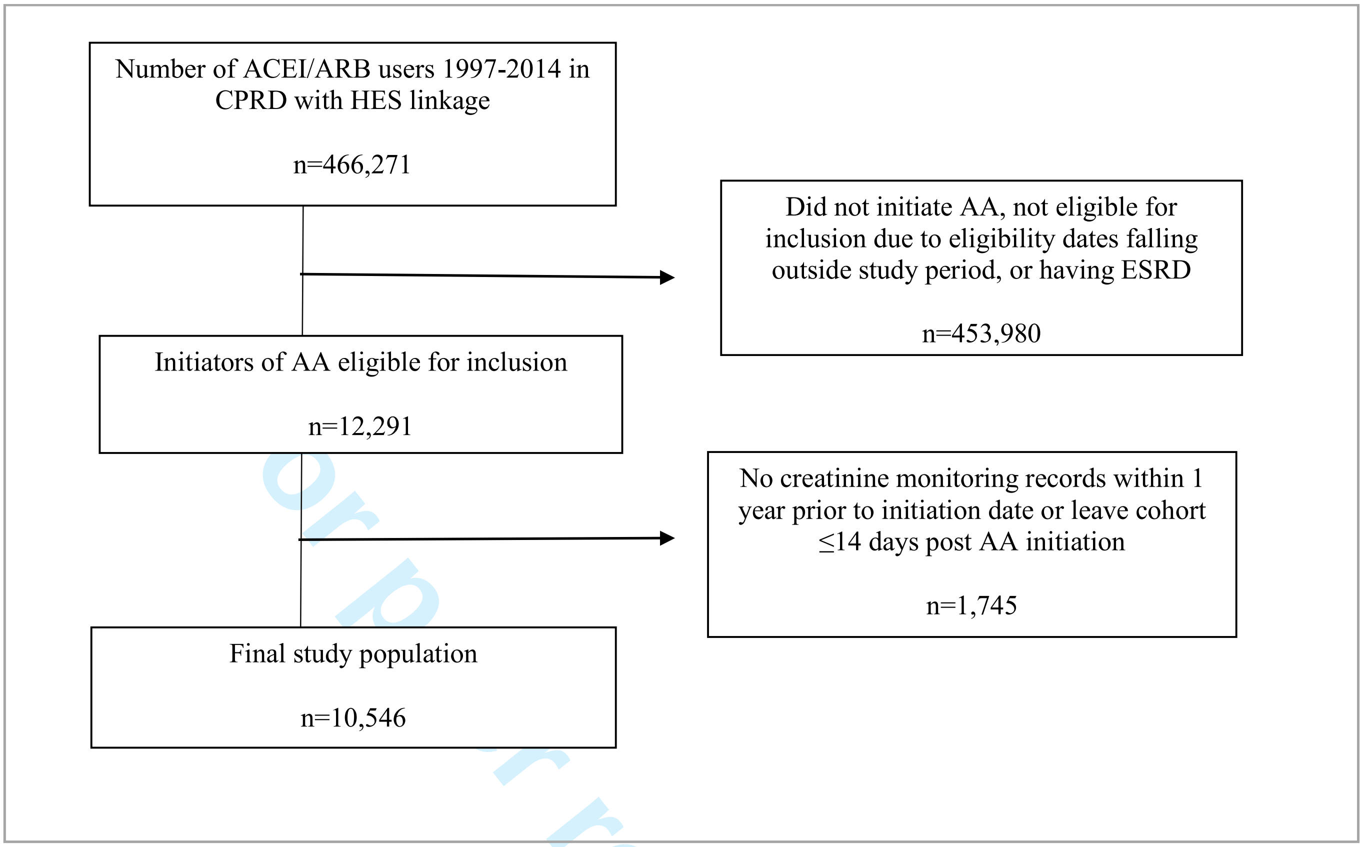

Figure 1: Flowchart demonstrating cohort selection

ACEI/ARB–angiotensin converting enzyme inhibitor/angiotensin receptor blocker, HES–hospital episode statistics, CPRD – Clinical Practice Research Datalink, AA-aldosterone antagonist, ESRD–end stage renal disease

For peer review only

Table 1: Characteristics of patients taking ACEI/ARB who initiate aldosterone antagonists in UK primary care 2004-2014, by monitoring groups

	Population	Monitoring ≤2 weeks	No monitoring ≤2 weeks
**Total number**	10546 (100)	3291 (100)	7255 (100)
**Female sex**	4348 (41.2)	1326 (40.3)	3022 (41.6)
**Age (years)**			
<50	656 (6.2)	138 (4.2)	518 (7.1)
50-59	1188 (11.3)	314 (9.5)	874 (12.1)
60-64	957 (9.1)	275 (8.4)	682 (9.4)
65-69	1227 (11.6)	391 (11.9)	836 (11.5)
70-74	1513 (14.4)	502 (15.3)	1011 (13.9)
75-79	1708 (16.2)	552 (16.8)	1156 (15.9)
≥80+	3297 (31.3)	1119 (34.0)	2178 (30.0)
**Ethnicity**			
White	4687 (44.4)	1476 (44.9)	3211 (44.3)
South Asian	119 (1.1)	33 (1.0)	86 (1.2)
Black	101 (1.0)	23 (0.7)	78 (1.1)
Mixed/Other	58 (0.6)	11 (0.3)	47 (0.7)
Missing/not stated	5581 (52.9)	1748 (53.1)	3833 (52.8)
**Smoking**			
Non-smoker	2693 (25.5)	835 (25.4)	1858 (25.6)
Current smoker	1505 (14.3)	405 (12.3)	1100 (15.2)
Ex-smoker	6333 (60.1)	2047 (62.2)	4286 (59.1)
Missing	15 (0.1)	~	11 (0.2)
**Body Mass Index (kg/m^2)**			
Underweight (<18.5)	191 (1.8)	69 (2.1)	122 (1.7)
Healthy weight (18.5 – 24.9)	2613 (24.8)	823 (25.0)	1790 (24.7)
Overweight (25 – 29.9)	3335 (31.6)	1020 (31.0)	2315 (31.9)
Obese (≥30)	4407 (41.8)	1379 (41.9)	3028 (41.7)
Missing	~	~	~
**Alcohol**			
Non-drinker	1268 (12.0)	360 (10.9)	908 (12.5)
Current drinker	7194 (68.2)	2308 (70.1)	4886 (67.4)
Ex-drinker	1451 (13.8)	447 (13.6)	1004 (13.8)
Missing	633 (6.0)	176 (5.4)	457 (6.3)
**Comorbidities**			
Hypertension	7928 (75.2)	2496 (75.8)	5432 (74.9)
IHD	6042 (57.3)	1919 (58.3)	4123 (56.8)
Heart Failure	6171 (58.5)	1983 (60.3)	4188 (57.7)
Arrhythmia	4495 (42.6)	1493 (45.4)	3002 (41.4)
Diabetes	3252 (30.8)	1043 (31.7)	2209 (30.5)
Peripheral Arterial Disease	946 (9.0)	326 (9.9)	6202 (8.6)
**Medication use**			
Calcium Channel Blocker	5043 (47.8)	1578 (48.0)	3465 (47.8)
Beta Blocker	6123 (58.1)	1976 (60.0)	4147 (57.2)
Thiazide diuretic	4354 (41.3)	1384 (42.1)	2970 (40.9)
Loop diuretic	7447 (70.6)	2490 (75.7)	4957 (68.3)
**eGFR category (ml/min/1.73m^2)[#]**			
≥60	5803 (55.0)	1665 (50.6)	4138 (57.0)
45-59	2668 (25.3)	864 (26.3)	1805 (24.9)
30-44	1666 (15.8)	591 (18.0)	1075 (14.8)
15-29	409 (3.9)	171 (5.2)	237 (3.3)
**Calendar time**			
2004-2006	2374 (22.5)	645 (19.6)	1729 (23.8)
2007-2009	3029 (28.7)	943 (28.7)	2086 (28.8)
2010-2014	5143 (48.8)	173 (51.8)	3440 (47.4)
**Biochemical parameters at baseline[‡]**			
Creatinine (µmol/L) (mean (SD))	99.1 (30.7)	102.7 (33.4)	97.4 (29.3)
Potassium (mmol/L) (mean (SD))	4.4 (0.7)	4.4 (0.9)	4.4 (0.6)
**Potassium (mmol/L)**			
<5.0mmol/L	8955(84.9)	2779 (84.4)	6176 (85.1)
5.0-5.5mmol/L	1136 (10.8)	367 (11.2)	769 (10.6)

For peer review only - http://bmjopen.bmj.com/site/about/guidelines.xhtml

For peer review only

>5.5mmo/L	252 (2.4)	78 (2.4)	174 (2.4)
Missing	203 (1.9)	67 (2.0)	136 (1.9)

Data shown are column number (%), except where mean (SD) is indicated.
~ refers to negligible.
IHD-Ischaemic Heart Disease; eGFR–estimated Glomerular Filtration Rate; AA-aldosterone antagonist; ACEI/ARB-angiotensin-converting enzyme inhibitor/angiotensin receptor blocker.
[#]Calculated from most recent creatinine measurement within 12 months before the first prescription date.
[‡]Baseline biochemical parameters were the values closest to AA initiation in the preceeding year.

Testing within two weeks of AA initiation

Within two weeks post AA initiation, 31.2% of the cohort had blood testing **(Table 1).** Approximately 64% of the population had testing within two months of initiation, while 95% had blood tests within one year **(Table 2)**. We conducted a sensitivity analysis among the 5,787 individuals not admitted to hospital within 30 days before or after AA initiation (54.9% of the whole cohort). Among these patients a similar proportion (32.7%) had follow up monitoring within two weeks **(Supplementary Table 1)**. The proportion of people having six tests within a year (roughly equating to optimal guideline recommended testing within 7 days, and in months 1,2,3,6 and 12) testing was approximately 1%.

Table 2: Proportion of patients taking ACEI/ARB who initiate aldosterone antagonists in UK primary care 2004-2014 with blood tests at several timepoints after initiation of AA

7 days	14 days	1 month	2 months	3 months	6 months	1 year
1674/10613	3291/10546	5210/10431	6520/10258	7229/10145	8389/9962	9294/9799
15.8%	31.2%	49.9%	63.5%	71.3%	84.2%	94.9%

The denominator changes due to patients exiting the cohort

Factors associated with testing within two weeks of AA initiation

Women and younger patients had lower odds for blood testing within two weeks of AA initiation **(Table 3)**. Those initiating AA after 2007 had higher odds of blood testing compared to patients initiating in 2004-2006**.** Those with high baseline potassium values did not have higer odds for receiving blood testing. No substantial differences were seen when the analysis was restricted to non-hospitalised patients although the increased odds of testing among patients with reduced baseline renal function was reduced, suggesting that there were disproportionate admissions among this group **(Supplementary Table 3)**.

For peer review only - http://bmjopen.bmj.com/site/about/guidelines.xhtml

Table 3: Association between patient characteristics and follow-up monitoring within two weeks following initiation of an aldosterone antagonist

	OR (95% CI)	
	Age and sex adjusted	Fully adjusted
**Male**	ref	ref
**Female**	0.90 (0.83 – 0.98)	0.90 (0.82 – 0.98)
**Age (years)**		
<50	0.54 (0.43 – 0.67)	0.58 (0.46 – 0.73)
50-59	0.72 (0.61 – 0.84)	0.78 (0.66 – 0.91)
60-64	0.81 (0.68 – 0.96)	0.86 (0.72 – 1.03)
65-69	0.94 (0.81 – 1.09)	0.96 (0.83 – 1.12)
70-75	ref	ref
76-79	0.90 (0.83-0.98)	0.95 (0.82 – 1.10)
80+	1.05 (0.92-1.20)	0.97 (0.85 – 1.11)
**eGFR category (ml/min/1.73m^2)**		
≥60	ref	ref
45-59	1.10 (0.99 – 1.23)	1.13 (1.01 – 1.27)
30-44	1.25 (1.10 – 1.42)	1.29 (1.14 - 1.46)
15-29	1.65 (1.33 – 2.06)	1.74 (1.40 - 2.16)
**Comorbidities**		
Hypertension	1.03 (0.93 – 1.13)	0.98 (0.88 – 1.09)
Ischaemic Heart Disease	1.00 (0.92 – 1.09)	0.98 (0.89 – 1.07)
Heart Failure	1.05 (0.96 – 1.15)	1.01 (0.92 – 1.11)
Arrhythmia	1.09 (1.00 – 1.19)	1.07 (0.98 – 1.18)
Diabetes	1.06 (0.97 – 1.16)	1.05 (0.95 – 1.15)
Peripheral Arterial Disease	1.12 (0.98 – 1.27)	1.08 (0.93 – 1.24)
**Calendar time**		
2004-2006	ref	ref
2007-2009	1.22 (1.07 – 1.38)	1.21 (1.07 – 1.38)
2010-2014	1.34 (1.17 – 1.53)	1.35 (1.18 – 1.55)
**Baseline potassium**		
<5.0mmol/L	ref	ref
5.0-5.5mmol/L	1.05 (0.91 – 1.20)	0.98 (0.86 – 1.12)
>5.5mmo/L	0.98 (0.73 – 1.30)	0.90 (0.68 -1.20)

eGFR–estimated Glomerular Filtration Rate.
Fully adjusted: adjusted for age, gender, CKD stage, hypertension, heart failure, ischaemic heart disease, arrhythmias, diabetes, peripheral arterial disease, baseline potassium and calendar time. Further adjustment for lifestyle covariates made marginal difference to all results, thus these variables are not included in models shown.

For peer review only

Adverse biochemical values

Of those with follow-up monitoring within two months of AA initiation (n=6,520), 2.0% had hyperkalaemia, 2.7% a creatinine ≥220µmol/L and 13.5% a 30% increase in creatinine from baseline, on their first blood test. **(Table 4)**. Of those with creatinine ≥220µmol/L, 29 people (16%) had creatinine ≥220µmol/L at baseline. A majority of patients with hyperkalaemia discontinued AA within 30 days of the blood test **(Table 4)**. However, less than one third of those with a >30% increase in creatinine discontinued AA, compared to 43% of those with a post-initiation creatinine ≥220µmol/L. Among non-hospitalised patients, the proportions with adverse laboratory values were marginally smaller while the proportion of people discontinuing the AA with each adverse biochemical value was similar **(Supplementary Table 4)**.

Table 4: Proportion with adverse biochemical values on testing within two months of AA initiation and number subsequently discontinuing aldosterone antagonist

	Hyperkalaemia (≥6mmol/L)[‡]	Creatinine ≥220µmol/L	≥30% increase in creatinine
Number with adverse biochemical values*	128/6373 (2.0%)	177/6520 (2.7%)	877/6520 (13.5%)
Number discontinuing AA[§]	68/128 (53.1%)	76/179 (42.9%)	251/877 (28.6%)

*Serum potassium and creatinine values on first blood test within two months of AA initiation.
[‡]Missing data for 147 people (2.25%) for first follow-up potassium value.
[§]Discontinuation defined as no further prescriptions of AA after blood test plus 30 days.
AA–aldosterone antagonist.

Factors associated with adverse biochemical values

In fully adjusted logistic regression models baseline eGFR<60mls/min/1.73m^2 and potassium ≥5mmol/L were independently associated with hyperkalaemia **(Table 5)**. Women had lower odds than men for a post-initiation creatinine ≥220µmol/L, but had a higher odds of a 30% increase in creatinine. There was no consistent evidence that baseline renal function was associated with developing a 30% increase in creatinine. In sensitivity analyses of the non-hospitalised cohort, similar associations between age and sex and the occurrence of adverse biochemical values were observed **(Supplementary Table 5)**.

For peer review only - http://bmjopen.bmj.com/site/about/guidelines.xhtml

Table 5: Associations between patient characteristics and adverse biochemical values after aldosterone antagonist initiation

	OR (95% CI)		
	Hyperkalaemia[‡*] (≥6mmol/L)	Creatinine[*] ≥220µmol/L	≥30% increase in creatinine[*]
**Female**	1.04 (0.70 – 1.54)	0.34 (0.23 – 0.51)	1.47 (1.25 – 1.73)
**Age (years)**			
<50	0.15 (0.02 – 1.08)	0.96 (0.32 – 2.84)	0.34 (0.21 – 0.55)
50-59	0.29 (0.10 – 0.86)	0.80 (0.32 – 2.84)	0.45 (0.32 – 0.65)
60-64	0.60 (0.28 – 1.31)	1.17 (0.53 – 2.56)	0.69 (0.49 – 0.96)
65-69	0.83 (0.43 – 1.59)	0.36 (0.16 – 0.83)	0.64 (0.49 – 0.85)
70-75	*ref*	*ref*	*ref*
76-79	0.75 (0.43 – 1.33)	0.71 (0.42 – 1.20)	0.94 (0.75 – 1.18)
80+	0.69 (0.40 – 1.19)	0.50 (0.30 – 0.83)	1.10 (0.87 – 1.39)
**eGFR category (ml/min/1.73m^2)**			
≥60	*ref*	*ref*	*ref*
45-59	2.06 (1.26 – 3.35)	2.80 (1.22 – 6.39)	0.81 (0.66 – 0.99)
30-44	2.32 (1.31 – 4.11)	20.83 (9.73 – 44.58)	0.82 (0.67 – 1.00)
15-29	3.62 (1.60 – 8.22)	248.0 (117.2-524.7)	1.01 (0.72 – 1.40)
**Comorbidities**			
Hypertension	1.27 (0.76 – 2.14)	0.93 (0.61 – 1.41)	1.18 (0.98 – 1.41)
Ischaemic Heart Disease	0.67 (0.45 – 1.00)	0.60 (0.43 – 0.85)	0.95 (0.82 – 1.11)
Heart Failure	0.77 (0.53 – 1.14)	0.97 (0.67 – 1.41)	1.12 (0.96 – 1.31)
Arrhythmia	1.00 (0.68 – 1.46)	0.81 (0.57 – 1.15)	1.11 (0.96 – 1.28)
Diabetes	1.21 (0.83 – 1.76)	1.11 (0.80 – 1.55)	1.18 (1.01 – 1.38)
Peripheral Arterial Disease	0.82 (0.44-1.51)	1.04 (0.64- 1.68)	1.09 (0.85 – 1.40)
**Calendar time**			
2004-2006	*ref*	*ref*	*ref*
2007-2009	1.14 (0.65-2.01)	1.36 (0.84 - 2.22)	0.97 (0.80 – 1.18)
2010-2014	1.08 (0.64- 1.83)	0.93 (0.58 – 1.51)	0.95 (0.78 – 1.15)
**Baseline potassium**			
<5.0mmol/L	*ref*		
5.0-5.5mmol/L	3.59 (2.43 - 5.32)	*n/a*	*n/a*
>5.5mmo/L	3.29 (1.57 – 6.88)		

*Adverse serum potassium and creatinine values on first blood test within two months of AA initiation.
N = 6,520 except ‡missing data for 147 people for first follow-up potassium value.
Only fully adjusted model is shown for clarity. Adjusted for age, gender, CKD stage, hypertension, heart failure, ischaemic heart disease, arrhythmias, diabetes, peripheral arterial disease, baseline potassium (hyperkalemia model only) and calendar time. Further adjustment for lifestyle covariates made marginal difference to all results and is not included.
eGFR–estimated Glomerular Filtration Rate.

For peer review only - http://bmjopen.bmj.com/site/about/guidelines.xhtml

DISCUSSION

In this cohort study of 10,546 users of ACEI/ARB, less than a third had follow up blood testing within two weeks of AA initiation. Two percent of the cohort developed severe hyperkalaemia within two months of AA initiation, and a similar proportion developed an absolute value of creatinine at which guidelines recommend AA cessation is considered. In the same time frame, 13.5% of the cohort developed a ≥30% increase in serum creatinine from baseline.

Strengths and Weaknesses

We used a linked primary and secondary care dataset to ensure complete information on comorbid diagnoses and to allow sensitivity analyses for those without hospital admissions. Further, our data represent a general population that is unrestricted by indication for therapy, thus our results are generalisable to the vast majority of patients receiving their treatment in primary care. We deliberately chose 2004 as the study start year to coincide with the roll out of the Quality and Outcomes Framework (QOF), a system of annual rewards to GPs which encouraged the standardisation of quality primary medical services. The introduction of QOF saw the incentivisation of creatinine testing amongst people with diabetes in 2004, and the establishment of a practice CKD register with the introduction of eGFR reporting in 2006.[21]

As well as examining the biochemical values of concern noted in current NICE practical guidelines for heart failure we also assessed the incidence of a ≥30% increase in creatinine after initiation of AA. This outcome is not discussed in the practical guidelines as a stopping criterion for AA initiation, but has been shown to have prognostic significance after initiation of ACEI/ARB treatment. [22]

However there are also limitations. Our results capture only those who had a record of blood testing in primary care and the risk of adverse events may be different in patients who were not tested. To maximise sample size and clinical relevance we included all patients with monitoring of creatinine. However approximately 2% of these patients had missing potassium values prior to AA initiation and 2.5% after AA initiation. We did not have access to blood test results from secondary care, which could have created a bias towards missing data among the sickest patients. However, restricting the analysis to individuals without any record of a hospitalisation in the 30-day period prior to and after AA initiation did not substantially change our results. The CKD-EPI equation for calculating eGFR requires an indicator for Afro-Caribbean ethnicity. Because ethnicity in our dataset was almost 50% missing, we did not use this variable in our calculations. We expect the impact of this to be small given that only 3% of the English population is of Afro-Caribbean ethnicity.[23]

For peer review only

Comparison with other studies

In a study of heart failure patients receiving health care through the Veterans Affairs health insurance programme in the US between 2003 and 2013, blood testing was carried out in 42% of patients within two weeks of initiation of AA.[9] Two other studies of heart failure patients carried out in North American settings found slightly higher monitoring rates for AA initiators; 53.5% within 7 days of initiation[3] and 44.6% within 10 days of initiation.[10] The contrast between our results and the results of North American research is noteworthy because it has been suggested in the past that monitoring in the UK is more frequent than in Canada.[4, 8] However, prior research on the frequency of monitoring in the UK was carried out in one geographical area and did not directly assess concordance with guideline recommended testing.[8]

Previous research examining patient characteristics associated with blood testing within 13 months of AA initiation found similar results to our study; that males, older patients and those with CKD had higher odds of receiving blood tests.[24] Diabetes has also been noted as a significant predictor of testing.[24] While diabetes was also weakly associated with testing in our study (OR 1.17 in sensitivity analysis of non-hospitalised patients), the association was less pronounced than in prior research (OR 1.63).[24]

We found that 2.0% of those initiating an AA experienced hyperkalaemia (potassium ≥6mmol/L) on the first blood testing event within two months of AA initiation. This compares to a rate of 2% found in RALES and 5.5% in the Eplerenone Post–Acute Myocardial Infarction Heart Failure Efficacy and Survival Study (EPHESUS), both clinical trials of AAs in patients with heart failure.[1, 25] In observational studies, rates of hyperkalaemia ranging from 6% [26] to 10% [27] have been reported. Such populations are said to be more reflective of the typical user of an AA in clinical practice than in clinical trials, and thus, the rates are thought to be more realistic.[4] The disparity between our results for hyperkalaemia and those found in other observational research may stem from our method of detecting potassium values only on the first blood test within two months , whereas prior studies examined peak potassium values within 3 months of initiation [26], and peak values within the individuals' entire treatment period.[27] As time since initiation increases it is increasingly likely that blood tests are performed due to an intercurrent illness or other event that may itself have contributed to hyperkalaemia or reduced renal function. Thus, we opted to look at the first blood testing event within two months to examine as much as possible a causal association between the initiation of an AA and the adverse biochemical value. In line with previous literature, we found that baseline potassium value was a significant predictor of hyperkalaemia after initiation of AA [25, 26, 28], as was reduced renal function.[24, 25, 26]

For peer review only - http://bmjopen.bmj.com/site/about/guidelines.xhtml

Using a threshold creatinine value as a safety indicator for renal function may not be an optimal measure given that absolute creatinine levels create an age, gender and ethnicity bias. Importantly we found that, consistent with a lower muscle mass, women had a lower odds of developing a creatinine ≥220µmmol post AA initiation in comparison to men but a 1.5 fold higher odds of a ≥30% increase in creatinine. This finding is particularly important given that women had a 9% lower odds of being tested within two weeks of AA initiation. The clinical implications of this are unknown but it is important to establish that there is not a discrepancy in related clinical outcomes. We have previously shown a greater risk of ≥30% change in creatinine after commencing ACEI/ARB, and asubsequently higher risk of acute kidney injury, among women compared to men.[14, 29] Increasing age was associated with increasing odds for ≥30% change in creatinine, even after adjusting for baseline renal function.[27] We found that approximately half of those with hyperkalaemia and creatinine ≥220µmol/L discontinued the AA within 30 days of the first post-initiation blood test which translates to an overall discontinuation rate of about 1%. This is lower than that reported in previous observational clinical research where approximately 7% of AA users required discontinuation for either hyperkalaemia or adverse renal events, although this was over the duration of therapy.[28]

Conclusions and clinical implications

We found that among patients taking an ACEI/ARB, less than one third of those initiating an AA received follow-up blood tests within two weeks. Of these, approximately 2% of patients developed potassium ≥6mmol/L or creatinine ≥220µmol/L on the first blood test within two months but 13.5% experienced a ≥30% increase in creatinine from baseline. Women had a 9% lower odds of having testing within two weeks, but had a higher odds of a ≥30% increase in creatinine than men. Baseline potassium levels >5.0mmol/L and baseline eGFR <60mls/min/1.73m^2 were independently associated with a potassium value ≥6mmol/L after AA initiation. Our results highlight the need for better adherence to monitoring guidelines, particularly for those in these high risk groups, and the importance of understanding the prognostic implications of changes in renal function after AA initiation.

For peer review only - http://bmjopen.bmj.com/site/about/guidelines.xhtml

DATA SHARING

No additional data avaialble

For peer review only

For peer review only - http://bmjopen.bmj.com/site/about/guidelines.xhtml

REFERENCES

1 Pitt B, Zannad F, Remme WJ, et al. The Effect of Spironolactone on Morbidity and Mortality in Patients with Severe Heart Failure. *New England Journal of Medicine* 1999;**341**:709-17.

2 Williams B, MacDonald TM, Morant S, et al. Spironolactone versus placebo, bisoprolol, and doxazosin to determine the optimal treatment for drug-resistant hypertension (PATHWAY-2): a randomised, double-blind, crossover trial. *The Lancet* 2015;**386**:2059-68.

3 Allen LA, Shetterly S, Peterson PN, et al. Guideline concordance of testing for hyperkalemia and kidney dysfunction during initiation of mineralocorticoid receptor antagonist therapy in patients with heart failure. *Circulation: Heart Failure* 2013;**7**:43-50.

4 Juurlink DN, Mamdani MM, Lee DS, et al. Rates of hyperkalemia after publication of the Randomized Aldactone Evaluation Study. *New England Journal of Medicine* 2004;**351**:543-51.

5 MHRA. Drug Safety Notice. Spironolactone and renin angiotensin system drugs in heart failure: risk of potentially fatal hyperkalaemia. Available at https://www.gov.uk/drug-safety-update/spironolactone-and-renin-angiotensin-system-drugs-in-heart-failure-risk-of-potentially-fatal-hyperkalaemia 2016.

6 National Institute for Health and Clinical Excellence (NICE). NICE Guideline CG108 Chronic Heart Failure. Appendix D – Practical Notes. Available at https://www.nice.org.uk/guidance/cg108/evidence/full-guideline-appendix-d-practical-notes-separate-file-136060532 2010.

7 National Institute for Health and Clinical Excellence (NICE). NICE Guideline CG127 Hypertension: Clinical management of primary hypertension in adults. 2011.

8 Wei L, Struthers AD, Fahey T, et al. Spironolactone use and renal toxicity: population based longitudinal analysis. *BMJ* 2010;**340**:c1768.

9 Chauhan V, Dev S, Pham M, et al. Facility variation and predictors of serum potassium monitoring after initiation of a mineralocorticoid receptor antagonist in patients with heart failure. *American heart journal* 2015;**170**:543-9.

10 Cooper LB, Hammill BG, Peterson ED, et al. Consistency of Laboratory Monitoring During Initiation of Mineralocorticoid Receptor Antagonist Therapy in Patients With Heart Failure. *JAMA* 2015;**314**:1973-5.

11 Herrett E, Gallagher AM, Bhaskaran K, et al. Data resource profile: clinical practice research datalink (CPRD). *International journal of epidemiology* 2015;**44**:827-36.

12 Herrett E, Thomas SL, Schoonen WM, et al. Validation and validity of diagnoses in the General Practice Research Database: a systematic review. *British journal of clinical pharmacology* 2010;**69**:4-14.

13 Herrett E, Shah AD, Boggon R, et al. Completeness and diagnostic validity of recording acute myocardial infarction events in primary care, hospital care, disease registry, and national mortality records: cohort study. *BMJ* 2013;**346**:f2350.

14 Schmidt M, Mansfield KE, Bhaskaran K, et al. Adherence to guidelines for creatinine and potassium monitoring and discontinuation following renin–angiotensin system blockade: a UK general practice-based cohort study. *BMJ Open* 2017;**7**:e012818.

15 Bhaskaran K, Douglas I, Forbes H, et al. Body-mass index and risk of 22 specific cancers: a population-based cohort study of 5· 24 million UK adults. *The Lancet* 2014;**384**:755-65.

16 Levey AS, Stevens LA, Schmid CH, et al. A New Equation to Estimate Glomerular Filtration Rate. *Annals of internal medicine* 2009;**150**:604-12.

17 Clase CM, Barzilay J, Gao P, et al. Acute change in glomerular filtration rate with inhibition of the renin-angiotensin system does not predict subsequent renal and cardiovascular outcomes. *Kidney International* 2016.

18 National Institute for Health and Clinical Excellence (NICE). NICE guideline CG182 - Chronic kidney disease in adults: assessment and management. 2014.

19 Mathur R, Bhaskaran K, Chaturvedi N, et al. Completeness and usability of ethnicity data in UK-based primary care and hospital databases. *Journal of public health* 2014;**36**:684-92.

For peer review only - http://bmjopen.bmj.com/site/about/guidelines.xhtml

20 StataCorp. Stata Statistical Software: Release 14. College Station, TX: StataCorp LP. 2015.
21 McDonald H. The epidemiology of infections among older people with diabetes mellitus and chronic kidney disease. London School of Hygiene & Tropical Medicine 2015.
22 Schmidt M, Mansfield KE, Bhaskaran K, et al. Serum creatinine elevation after renin-angiotensin system blockade and long term cardiorenal risks: cohort study. *BMJ* 2017;**356**:j791.
23 Office for National Statistics. Census: Key Statistics for local authorities in England and Wales, table number KS201EW [online]. 2012. Available from: www.ons.gov.uk/Fons/Frel/Fcensus/F2011-census/Fkey-statistics-for-local-authorities-in-england-and-wales/Frft-table-ks201ew.xls. 2012.
24 Raebel MA, McClure DL, Chan KA, et al. Laboratory evaluation of potassium and creatinine among ambulatory patients prescribed spironolactone: are we monitoring for hyperkalemia? *Annals of Pharmacotherapy* 2007;**41**:193-200.
25 Pitt B, Bakris G, Ruilope LM, et al. Serum Potassium and Clinical Outcomes in the Eplerenone Post–Acute Myocardial Infarction Heart Failure Efficacy and Survival Study (EPHESUS). *Circulation* 2008;**118**:1643-50.
26 Shah KB, Rao K, Sawyer R, et al. The adequacy of laboratory monitoring in patients treated with spironolactone for congestive heart failure. *Journal of the American College of Cardiology* 2005;**46**:845-9.
27 Svensson M, Gustafsson F, Galatius S, et al. How prevalent is hyperkalemia and renal dysfunction during treatment with spironolactone in patients with congestive heart failure? *Journal of cardiac failure* 2004;**10**:297-303.
28 Tamirisa KP, Aaronson KD, Koelling TM. Spironolactone-induced renal insufficiency and hyperkalemia in patients with heart failure. *American heart journal* 2004;**148**:971-8.
29 Mansfield KE, Nitsch D, Smeeth L, et al. Prescription of renin–angiotensin system blockers and risk of acute kidney injury: a population-based cohort study. *BMJ open* 2016;**6**:e012690.

For peer review only

For peer review only - http://bmjopen.bmj.com/site/about/guidelines.xhtml

Supplementary Information

SI Table 1: Proportion of non-hospitalised patients taking ACEI/ARB who initiate aldosterone antagonists in UK primary care 2004-2014 with blood tests with 14 days of AA initiation

Non-hospitalised population	Monitoring ≤14 days post initiation (n., %)	No monitoring ≤14 days post initiation (n., %)
n=5787	1892 (32.7)	3897 (67.3)

Proportions for monitoring use 5787 as denominator. Non-hospitalisation refers to not having a hospitalisation in the 30 days prior to or post initiation of AA.

For peer review only

SI Table S2: Summary of clinical guideline recommendations for blood testing after initiation of AA

	NICE HF*	NICE HF Practical Guidelines Appendix D	NICE HF Practical Guidelines Appendix M	NICE Hypertension§	British National Formulary	American Guidelines∞	European Guidelines
≤7 days	*na*	x	x	*na*	x	x	x
≤1 month	*na*	x	x	x	X	x	x
≤2 month	*na*	x	x	*na*	X	x	x
≤3 month	*na*	x	x	*na*	X	x	x
≤6 month	*na*	x	x	*na*	X	x	x
≤9 month	*na*	*na*	x	*na*	X	x	x
≤12 month	*na*	x	x	*na*	X	x	x
How frequently after 1st year	*na*	6 monthly	6 monthly	*na*	6 monthly	3 monthly	4 monthly
Discontinue if K≥6mmol/L	*na*	x	x	*na*	*na*	x∞∞	x
Discontinue if creatinine ≥220µmol/L	*na*	x	x†	*na*	*na*	*na***	x‡
Discontinue if relative changes between baseline creatinine and follow up creatinine or eGFR ≥X%	*na*	*na*	*na*	*na*	*na*	*na***	*na*

* NICE HF guidelines recommend monitoring within two weeks after a medication change, but refer to Appendix D for guidance on monitoring for hyperkalaemia and renal function deterioration

† Recommends discontinuing if creatinine ≥200µmol/L

§ Recommends blood testing as required after testing within one month of AA initiation/increased diuretic use

∞ Recommends blood testing within 2-3 days of AA initiation, and again at 7 days.

∞∞ Recommends discontinuation at potassium > 5.5mEq/L

** Does not provide strict stopping/starting criteria according to serum creatinine thresholds. Suggests that risk of hyperkalaemia increases with worsening renal function and in elderly patients, or patients with low muscle mass in whom serum creatinine does not accurately reflect GFR, determination that GFR is >30ml/min/1.73m^2 is recommended.

‡ Recommends discontinuing if creatinine ≥310µmol/L or eGFR <20ml/min/1.73m^2

European Guidelines: “2016 ESC Guidelines for the diagnosis and treatment of acute and chronic heart failure”

For peer review only

American Guidelines: “2013 ACCF/AHA Guideline for the Management of Heart Failure”
NICE: National Institute for Health and Care Excellence
HF: Heart Failure

For peer review only

SI Table 3: Association between non-hospitalised patient characteristics and follow up monitoring within two weeks of initiation of an aldosterone antagonist

	OR (95% CI)	
	**Age and sex adjusted**	**Fully adjusted**
**Male**	*ref*	*ref*
**Female**	0.88 (0.78 – 0.99)	0.91 (0.80 – 1.03)
**Age (years)**		
<50	0.38 (0.28 – 0.53)	0.42 (0.30 – 0.58)
50-59	0.69 (0.56 – 0.85)	0.75 (0.60 – 0.93)
60-64	0.83 (0.66 – 1.06)	0.89 (0.70 – 1.13)
65-69	0.93 (0.76 – 1.14)	0.95 (0.77 – 1.17)
70-75	*ref*	*ref*
76-79	0.91 (0.75 – 1.11)	0.92 (0.75 – 1.12)
80+	1.13 (0.95 – 1.34)	1.11 (0.92 – 1.32)
**eGFR category (ml/min/1.73m^2)**		
≥60	*ref*	*ref*
45-59	1.00 (0.86 – 1.16)	1.02 (0.87 – 1.19)
30-44	1.10 (0.93 – 1.31)	1.10 (0.92 – 1.32)
15-29	1.13 (0.82 – 1.57)	1.15 (0.83 – 1.60)
**Comorbidities**		
Hypertension	0.98 (0.86 – 1.12)	0.96 (0.83 – 1.60)
Ischaemic Heart Disease	1.09 (0.97 – 1.23)	1.04 (0.93 – 1.17)
Heart Failure	1.17 (1.04 – 1.32)	1.11 (0.97 – 1.26)
Arrhythmia	1.16 (1.03 – 1.31)	1.10 (0.97 – 1.25)
Diabetes	1.16 (1.02 – 1.33)	1.17 (1.02 – 1.34)
Peripheral Arterial Disease	1.13 (0.93 – 1.38)	1.06 (0.87 – 1.31)
**Calendar time**		
2004-2006	*ref*	*ref*
2007-2009	1.26 (1.07 – 1.49)	1.25 (1.06 – 1.49)
2010-2014	1.36 (1.15 – 1.61)	1.35 (1.13 – 1.60)
**Baseline potassium**		
<5.0mmol/L	*ref*	*ref*
5.0-5.5mmol/L	1.05 (0.87 – 1.27)	1.01 (0.84 – 1.23)
>5.5mmo/L	0.87 (0.59 – 1.30)	0.85 (0.57 – 1.26)

eGFR–estimated Glomerular Filtration Rate.
Fully adjusted: adjusted for age, gender, CKD stage, hypertension, heart failure, ischaemic heart disease, arrhythmias, diabetes, peripheral arterial disease, baseline potassium and calendar time. Further adjustment for lifestyle covariates and socioeconomic status made marginal difference to all results, thus these variables are not included in models shown.

For peer review only

SI Table 4: Proportion of non-hospitalised patients with adverse biochemical values on testing within 2 months of AA initiation and number subsequently discontinuing aldosterone antagonist

	Hyperkalaemia (>=6mmol/L)[‡]	Creatinine (≥220µmol/L)	≥30% Change in Creatinine
Number with adverse biochemical values (n,%)*	60/3698 (1.6%)	75/3757 (2.0%)	374/3757 (10.0%)
Number discontinuing AA (n, %)[∞]	30/60 (50.0%)	29/75 (38.7%)	109/374 (29.1%)

*serum potassium and creatinine values on first blood test within two months of AA initiation.
[‡]Missing data for 59 people for first follow-up potassium value.
[§]Discontinuation defined as no further prescriptions of AA after blood test plus 30 days.
AA–aldosterone antagonist.

For peer review only

SI Table 5: Associations between non-hospitalised patient characteristics and adverse biochemical values after aldosterone antagonist initiation

	OR (95% CI)		
	**Hyperkalaemia* (>=6mmol/L)**	**Creatinine* (≥220μmol/L)**	**≥30% Change in Creatinine**
**Male**	*ref*	*ref*	*ref*
**Female**	1.53 (0.93 – 2.51)	0.78 (0.50 – 1.23)	1.52 (1.22 – 1.90)
**Age (years)**			
<50	-	0.80 (0.22 – 2.87)	0.37 (0.16 – 0.85)
50-59	0.47 (0.15 – 1.45)	0.45 (0.16 – 1.30)	0.67 (0.40 – 1.13)
60-64	0.57 (0.18 – 1.79)	0.67 (0.23 – 1.94)	0.79 (0.47 – 1.30)
65-69	0.41 (0.13 – 1.28)	0.20 (0.05 – 0.81)	0.81 (0.52 – 1.26)
70-75	*ref*	*ref*	*ref*
76-79	0.93 (0.44 - 1.97)	1.34 (0.65 – 2.78)	1.05 (0.73 – 1.51)
80+	0.82 (0.41 - 1.64)	1.22 (0.63 – 2.37)	1.25 (0.87 – 1.80)
**eGFR category (ml/min/1.73m^2)**			
≥60	*ref*	*ref*	*ref*
45-59	3.36 (1.40 – 8.05)	5.02 (1.24 – 20.24)	0.84 (0.64 – 1.10)

For peer review only

30-44	6.01 (2.42 – 14.89)	47.79 (13.70 – 166.76)	1.11 (0.81 – 1.53)
15-29	8.69 (2.51 – 30.10)	875.60 (240.46 – 3188.32)	1.23 (0.75 – 2.04)
**Comorbidities**			
Hypertension	1.11 (0.51 – 2.37)	1.12 (0.60 -2.08)	1.30 (0.98 - 1.73)
Ischaemic Heart Disease	0.52 (0.28 – 0.96)	0.59 (0.33 – 1.07)	1.03 (0.84 -1.27)
Heart Failure	0.72 (0.42 – 1.22)	0.98 (0.55 -1.73)	0.92 (0.73 - 1.15)
Arrhythmia	0.93 (0.54 – 1.61)	0.57 (032 – 1.01)	1.03 (0.82 -1.29)
Diabetes	0.96 (0.53 – 1.74)	1.10 (0.61 – 1.98)	0.97 (0.76 – 1.24)
Peripheral Arterial Disease	1.05 (0.38 – 2.87)	0.55 (0.21 – 1.40)	0.89 (0.59 - 1.34)
**Calendar time**			
2004-2006	*ref*	*ref*	*ref*
2007-2009	0.94 (0.42 – 2.07)	1.19 (0.60 – 2.38)	1.01 (0.75 – 1.35)
2010-2014	0.91 (0.46 – 1.77)	0.76 (0.40 – 1.47)	0.96 (0.72 – 1.27)
**Baseline potassium**			
<5.0mmol/L			
5.0-5.5mmol/L	2.52 (1.31 – 4.83)	*n/a*	*n/a*
>5.5mmo/L	5.26 (1.87 – 14.81)		

*Adverse serum potassium and creatinine values on first blood test within two months of AA initiation.
N = 3,757 except missing data for 59 people for first follow-up potassium value.
Only fully adjusted model is shown for clarity. Adjusted for age, gender, CKD stage, hypertension, heart failure, ischaemic heart disease, arrhythmias, diabetes, peripheral arterial disease, baseline potassium (hyperkalemia model only) and calendar time. Further adjustment for lifestyle covariates and socioeconomic status made marginal difference to all results and is not presented.
eGFR–estimated Glomerular Filtration Rate.

For peer review only

Laboratory monitoring after initiation of aldosterone antagonist therapy in users of renin-angiotensin system blockers: A UK primary care cohort study

Journal:	*BMJ Open*
Manuscript ID	bmjopen-2017-018153.R1
Article Type:	Research
Date Submitted by the Author:	06-Sep-2017
Complete List of Authors:	Sinnott, Sarah-Jo; London School of Hygiene and Tropical Medicine, Non-communicable disease epidemiology<br>Mansfield, Kathryn; London School of Hygiene and Tropical Medicine, Non-communicable disease epidemiology<br>Schmidt, Morten; London School of Hygiene and Tropical Medicine, Non-communicable disease epidemiology; Aarhus Universitet, Dept of Clinical Epidemiology<br>Bhaskaran, Krishnan; London School of Hygiene and Tropical Medicine, Non-communicable disease epidemiology<br>Smeeth, Liam; London School of Hygiene and Tropical Medicine, Non-communicable disease epidemiology<br>Nitsch, Dorothea; London School of Hygiene and Tropical Medicine, Non-communicable disease epidemiology<br>Tomlinson, Laurie; London School of Hygiene and Tropical Medicine, Non-communicable disease epidemiology
**Primary Subject Heading**:	Cardiovascular medicine
Secondary Subject Heading:	Cardiovascular medicine, Epidemiology, Health services research, Pharmacology and therapeutics
Keywords:	drug monitoring, Heart failure < CARDIOLOGY, Hypertension < CARDIOLOGY, electronic health records, health care delivery

SCHOLARONE™ Manuscripts

Laboratory monitoring after initiation of aldosterone antagonist therapy in users of renin-angiotensin system blockers: A UK primary care cohort study.

Authors:

Sarah-Jo Sinnott[1], Kathryn E Mansfield[1], Morten Schmidt[1,2,3] Krishnan Bhaskaran[1], Liam Smeeth[1], Dorothea Nitsch[1] Laurie A Tomlinson[1]

1. Department of non-communicable disease epidemiology, London School of Hygiene and Tropical Medicine, Keppel St, London, WC1E7HT
2. Department of Clinical Epidemiology, Aarhus University Hospital, Denmark
3. Department of Cardiology, Regional Hospital West Jutland, Herning, Denmark

Corresponding Author:

Sarah-Jo Sinnott
Department of non-communicable disease epidemiology, London School of Hygiene & Tropical Medicine, Keppel St, London, WC1E7HT
Email: sarah-jo.sinnott@lshtm.ac.uk
Phone 02072994821

Word count

~3400

Abstract count

199

Tables and figures

1 Figure

5 Tables

Keywords

Drug monitoring, heart failure, hypertension, electronic medical records, health care delivery.

For peer review only - http://bmjopen.bmj.com/site/about/guidelines.xhtml

For peer review only

COPYRIGHT/LICENCSE FOR PUBLICATION

"The Corresponding Author has the right to grant on behalf of all authors and does grant on behalf of all authors, an exclusive licence (or non exclusive for government employees) on a worldwide basis to the BMJ Publishing Group Ltd to permit this article (if accepted) to be published in BMJ editions and any other BMJPGL products and sublicences such use and exploit all subsidiary rights, as set out in our licence."

CONTRIBUTORSHIP

LAT had the idea for the study and acquired data permissions. SJS, MS, KEM, DN and LAT designed the study. SJS, KEM and MS managed the data and established the cohort. SJS did the analyses. All authors (SJS, KEM, MS, KB, LS, DN, LAT) participated in the discussion and interpretation of the results. SJS organised the writing and wrote the initial drafts. All authors (SJS, KEM, MS, KB, LS, DN, LAT) critically revised the manuscript for intellectual content and approved the final version. SJS is the guarantor.

TRANSPARENCY

SJS affirms that the manuscript is an honest, accurate, and transparent account of the study being reported; that no important aspects of the study have been omitted; and that any discrepancies from the study as planned have been explained.

ETHICS

The protocol for this study was approved by London School of Hygiene and Tropical Medicine Ethics Committee (No. 6536) and the Independent Scientific Advisory Committee (ISAC) for Medicines and Healthcare Products Regulatory Agency (No. 16_025A).

FUNDING

SJS is supported by a Wellcome Trust Sir Henry Wellcome Fellowship [107340/Z/15/Z]. LS is supported by a Wellcome Trust Senior Research Fellowship in Clinical Science [098504/Z/12/Z]. LAT and KEM are supported by a Wellcome Trust Intermediate Clinical Fellowship to LAT [101143/Z/13/Z]. KB is supported by a Sir Henry Dale Fellowship jointly funded by the Wellcome Trust and the Royal Society [107731/Z/15/Z]. MS was supported by the A.P. Møller Foundation for the Advancement of Medical Science, Snedkermester Sophus Jacobsen & Hustru Astrid Jacobsens Fond, and Christian og Ottilia Brorsons Rejselegat for yngre videnskabsmænd og–kvinder.

COMEPETING INTERESTS

All authors have completed the ICMJE uniform disclosure form at www.icmje.org/coi_disclosure.pdf and declare: SJS, KEM, MS, DN and LAT nothing to declare; LS reports grants from Wellcome Trust and British Heart Foundation during the conduct of the study, grants from Wellcome Trust, Medical Research Council, National Institute for Health Research and the European Union outside the submitted work, personal fees from GSK for advisory work unrelated to the submitted work, grant funding from GSK for academic research unrelated to the submitted work, acts as an unpaid steering committee chair for AstraZeneca for a randomised trial unrelated to the submitted work, and is a trustee of the British Heart Foundation. KB declares grants from the Wellcome Trust, Royal Society, MRC, NIHR and BHF outside the submitted work.

For peer review only - http://bmjopen.bmj.com/site/about/guidelines.xhtml

ABSTRACT

Objective

To determine the frequency of biochemical monitoring after initiation of aldosterone atagonists, in patients also using angiotensin-converting enzyme inhibitor/angiotensin receptor blockers (ACEI/ARB).

Setting

UK primary care

Participants

ACEI/ARB users who initiated an AA between 2004-2014.

Outcomes

We calculated the proportions with: 1) biochemical testing ≤2 weeks post AA initiation; 2) adverse biochemical values ≤2 months (potassium ≥6mmol/L; creatinine ≥220µmol/L; ≥30% increase in creatinine from baseline); 3) discontinuers of AA in those with an adverse biochemical value. We used logistic regression to study patient characteristics associated with testing and adverse biochemical values.

Results

In 10,546 initiators of AA, 3,291 (31.2%) had a record of testing ≤2 weeks post initiation. A total of 2.0% and 2.7% of those with follow-up testing within2 months of initiation experienced potassium ≥6mmol/L and creatinine ≥220µmol/L respectively, while 13.5% had a ≥30% increase in creatinine. Baseline potassium (OR 3.59, 95% CI 2.43-5.32 for 5.0-5.5mmol/L compared to <5.0mmol/L) and eGFR 45-59mls/min/1.73m^2 (OR 2.06, 95% CI 1.26–3.35 compared to ≥60mls/min/1.73m^2) were independently predictive of potassium ≥6mmol/L. Women and people with diabetes had higher odds of ≥30% increase in creatinine.

Conclusion

Less than one-third of patients taking ACEI/ARB had testing within 2 weeks of initiating aldosterone antagonists.

For peer review only - http://bmjopen.bmj.com/site/about/guidelines.xhtml

STRENGTHS AND LIMITATIONS OF THE STUDY

- This is a population cohort study, based on electronic health records from UK primary care, examining whether users of renin-agiotensin system blockade who commence aldosterone antagonists (AA) have appropriate biochemical monitoring after initiation of AA.
- The population was not restricted by indication for therapy.
- Those who were hospitalised prior to or immediately after initiating an AA may have had missing data for test results in primary care data. In a sensitivity analysis, we used primary care data linked to hospital data to assess monitoring in a population that was not hospitalised. We found similar rates of monitoring and adverse events compared to the main analysis.

For peer review only

INTRODUCTION

Angiotensin-converting enzyme inhibitors or angiotensin receptor blockers (ACEI/ARB) and aldosterone antagonists (AA), such as spironolactone and eplenerone, are frequently used in combination. They provide reductions in morbidity and mortality for patients with heart failure [1] and reductions in blood pressure for patients with resistant hypertension.[2] However, users of these drugs are at risk of acute kidney injury [3], hyperkalaemia, and hyperkalaemia-associated mortality.[4]

The occurrence of adverse events associated with combined ACEI/ARB and AA use was recently highlighted in the UK with the publication of a drug safety notice from the Medicines and Healthcare products Regulatory Agency (MHRA). It reported on the increasing incidence of life-threatening hyperkalaemic adverse events in patients prescribed ACEI/ARB and spironolactone.[5]

To help avoid adverse events after initiation of AA, biochemical parameters should be monitored.[6] At present, the National Institute of Health and Care Excellence (NICE) practical guidelines for heart failure recommend testing for potassium, creatinine, and estimated Glomerular Filtration Rate (eGFR) after one week, and at one, two, three, and six months, and six monthly thereafter, following initiation of an AA in heart failure.[7] These guidelines recommend stopping the AA if potassium ≥6mmol/L and if creatinine ≥220µmol/L.[7] NICE guidelines for hypertension state that testing for sodium, potassium and renal function should occur within one month after inititiation of AA, and as required thereafter.[8]

It is not known how well these guidelines are adhered to in the UK. Previous evidence on blood testing during AA treatment in the UK is historic, restricted to one geographical region, and did not specifically assess adherence to guideline recommended blood testing.[9] Data from North America suggest that recommended blood testing occurs in less than 50% of patients.[3, 10, 11] Poor monitoring of patients taking these drug combinations, as well as increasing use among patients at high risk of adverse outcomes, may help to explain the increased occurrence of hyperkalaemic events as reported by the MHRA.

Therefore, among a large, recent cohort of users of ACEI/ARB who initiated an AA, we sought to examine patterns of blood testing, and the occurrence of hyperkalaemia and renal impairment. Our aims were to determine: 1) the proportion of people initiating an AA who had testing with two weeks of initiation; 2) the patient characteristics associated with testing; 3) the proportion of people who had adverse biochemical values after initiation of AA, and the proportion that then discontinued the AA and; 4) the patient characteristics associated with adverse biochemical values.

For peer review only - http://bmjopen.bmj.com/site/about/guidelines.xhtml

METHODS

Data

The Clinical Practice Research Database (CPRD-GOLD) is a nationally representative repository of de-identified electronic medical records from primary care in the UK. It holds data on demographics, health related behaviours, test results, diagnoses, referrals and prescriptions for 4.4 million people currently alive with research-quality data.[12] It is one of the largest databases of longitudinal medical records from primary care globally and has been extensively validated.[12, 13] For this study, data on test results were extracted from these primary care records.

For this study, we used CPRD-GOLD data linked to Hospital Episodes Statistics (HES). This linkage is possible for 60% of English practices contributing to the CPRD-GOLD database. The HES database provides data on the primary diagnosis for a hospital admission, as well as other diagnoses and procedures carried out during that admission. The linkage thus provides a more complete picture of comorbidities, improves the accuracy of timing and in this study allowed the conduct of sensitivity analyses in those without hospital admissions.[14]

Population

We identified a cohort of HES-linked CPRD patients aged ≥18 years, who initiated ACEI/ARB treatment between April 1, 1997 and March 31, 2014.[15] We identified continuous courses of ACEI/ARB therapy by allowing for a 90-day gap between the end date of one prescription and the start of the next consecutive prescription (to allow for stock piling and medications prescribed in secondary care). Among this cohort, we identified people who subsequently became new users of AA in the period 2004-2014. New use of AA was defined as no use of AA in the year prior to first prescription. We then restricted the population to those with a record for creatinine monitoring in the year prior to AA initiation. We assumed that those with creatinine test results prior to and after AA initiation were also tested for potassium. If values for potassium were not present they were treated as missing. This approach avoided exclusion of patients whose blood sample may have been haemolysed, resulting in potassium value not reported. Patients with READ codes for end-stage renal disease and estimated glomerular filtration rates (eGFR) values corresponding with chronic kidney disease stage 5 prior to cohort entry were excluded. Patients were eligible for follow up until the earliest of; death, transfer out of practice, last data collection or end of the study (March 2014).

Covariates

We obtained information for all patients on age, gender, calendar time of AA initiation (2004-2006, 2007-2009, 2010-2014), and lifestyle factors. The closest records to AA initiation date were used for

For peer review only - http://bmjopen.bmj.com/site/about/guidelines.xhtml

determining smoking, alcohol and body mass index status using existing algorithms.[16] Ethnicity was categorised as white, black, south Asian, and other/mixed. We extracted data on the cardiovascular comorbidities and diabetes using data from both CPRD and HES. We calculated baseline eGFR using the most recent creatinine value from CPRD data prior to AA initiation and the Chronic Kidney Disease Epidemiology Collaboration (CKD-EPI) equation.[17] CPRD prescribing data were used to extract data on baseline medication use. Baseline potassium values were categorised as: <5mmol/L, 5–5.5mmol/L and >5.5mmol/L.

Outcomes and Statistical Analyses

Several guidelines recommend time periods for blood testing after initiation of an AA **(Supplementary Table 1)**. We assessed whether monitoring occurs in line with current NICE recommendations, with some modification. NICE practical guidelines for heart failure recommend blood testing within one week after AA initiation.[7] However, we calculated the proportion of AA initiators who had blood testing within two weeks of AA initiation to accommodate the practical challenges of immediate follow-up testing faced in clinical practice. In additional analyses, we also calculated the proportion of people who had testing 1) within seven days post AA initiation, 2) within one month, two months, 3 months, 6 months and 12 months post AA initiation and 3) on all recommended monitoring occasions.[3] Guidelines offer a framework for clinicians on how to treat and manage patients but adherence to guidelines is not obligatory and deviations may just reflect individualised care. Nonetheless, using clinical guidelines to frame this analysis provides a series of time points against which we can quantify frequency of monitoring for patients using combinations of AA and ACEI/ARB.

We used thresholds set out by the NICE practical guidelines for heart failure to calculate the proportion of patients who had an adverse biochemical value on their first blood test within two months of initiation.[7] Hyperkalaemia was a potassium of ≥6mmol/L and an adverse creatinine value indicating renal dysfunction was defined as creatinine ≥220µmol/L. We chose two months to accord with monitoring periods in previous clinical trials[18], and also because at the outset we expected that a majority of patients would have testing within this timeframe. We then calculated the proportion of those with adverse biochemical values who discontinued the AA. We used a conservative definition of discontinuation, to prevent misclassification of people who had blood tests at the beginning of a median length prescription (28 days). Therefore, we classified discontinuation as no further prescription of an AA beyond 30 days after the first post-initiation blood test, i.e., when the end date of the course of AA therapy occurred before the first blood test date plus 30 days.

For peer review only - http://bmjopen.bmj.com/site/about/guidelines.xhtml

Some practical guidelines for initiation of AA refer to an absolute level of creatinine to indicate renal dysfunction of concern when stopping the AA should be considered (**Supplementary Table 1)**. However, absolute values of creatinine reflect substantially different levels of eGFR depending on the age, gender and ethnicity of the patient. A proportional change in creatinine or eGFR is recommended by NICE guidelines to indicate significant change in renal function for patients who initiate ACEI/ARB.[19] This measure is therefore clinically familiar and more closely indicates changes in renal function that may be associated with a new drug. Therefore, we also calculated the number of people who experienced a 30% relative increase in creatinine from baseline as an adverse biochemical finding. We used the most recent values for creatinine within one year prior to AA initiation and creatinine values on the first blood test within two months post AA initiation to calculate the relative change.

To assess patient level characteristics associated with testing within two weeks (versus not having testing within two weeks) we used logistic regression with robust standard errors to adjust for correlations between patients within practices.[10] The crude model adjusted for age and gender only, while the fully adjusted model adjusted for age, gender, eGFR category, cardiovascular comorbidities, diabetes, baseline potassium and calendar time. We did not include ethnicity in fully adjusted models due to missing data for approximately 50% of the population.[20] We also used logistic regression to assess patient characteristics associated with an adverse biochemical value (versus not having an adverse biochemical value), with adjustments for the same variables as in the prior model.

In a sensitivity analysis, we assessed the proportion of patients receiving blood tests among patients who were not hospitalised in the 30-day period prior to or after AA initiation. Data on laboratory tests are not available in the HES database, and test results may not always be sent from the hospital to the GP practice. Therefore, this analysis excluded patients who had blood tests as inpatients , which would contribute to an apparently low observed level of testing in primary care.

We used Stata version 14 for all analyses.[21]

The protocol for this study was approved by London School of Hygiene and Tropical Medicine Ethics Committee (No. 6536) and the Independent Scientific Advisory Committee (ISAC) for Medicines and Healthcare Products Regulatory Agency (No. 16_025A).

For peer review only - http://bmjopen.bmj.com/site/about/guidelines.xhtml

RESULTS

From 466,271 continuous users of ACEI/ARB between 1997 and March 2014, 12,291 people initiated an AA between 2004-2014 and were eligible for inclusion. After further exclusions, 10,546 were ultimately included in the cohort. **(Figure 1)**. The population was 41% female and had a mean age of 71.8 years (SD 12.9) **(Table 1)**. Mean baseline serum potassium was 4.4 mmol/L (SD 0.7). Approximately one fifth of the population had eGFR <30mls/min/1.73m^2 **(Table 1).** Spironolactone was the drug commenced for 9,917/10,546 (94%) of those initiating an AA, with the remainder initiating eplenerone.

Insert Figure 1 here

For peer review only

For peer review only - http://bmjopen.bmj.com/site/about/guidelines.xhtml

Table 1: Characteristics of patients taking ACEI/ARB who initiate aldosterone antagonists in UK primary care 2004-2014, by monitoring groups

	Population	Monitoring ≤2 weeks	No monitoring ≤2 weeks
**Total number**	10546 (100)	3291 (100)	7255 (100)
**Female sex**	4348 (41.2)	1326 (40.3)	3022 (41.6)
**Age (years)**			
<50	656 (6.2)	138 (4.2)	518 (7.1)
50-59	1188 (11.3)	314 (9.5)	874 (12.1)
60-64	957 (9.1)	275 (8.4)	682 (9.4)
65-69	1227 (11.6)	391 (11.9)	836 (11.5)
70-74	1513 (14.4)	502 (15.3)	1011 (13.9)
75-79	1708 (16.2)	552 (16.8)	1156 (15.9)
≥80+	3297 (31.3)	1119 (34.0)	2178 (30.0)
**Ethnicity**			
White	4687 (44.4)	1476 (44.9)	3211 (44.3)
South Asian	119 (1.1)	33 (1.0)	86 (1.2)
Black	101 (1.0)	23 (0.7)	78 (1.1)
Mixed/Other	58 (0.6)	11 (0.3)	47 (0.7)
Missing/not stated	5581 (52.9)	1748 (53.1)	3833 (52.8)
**Smoking**			
Non-smoker	2693 (25.5)	835 (25.4)	1858 (25.6)
Current smoker	1505 (14.3)	405 (12.3)	1100 (15.2)
Ex-smoker	6333 (60.1)	2047 (62.2)	4286 (59.1)
Missing	15 (0.1)	~	11 (0.2)
**Body Mass Index (kg/m^2)**			
Underweight (<18.5)	191 (1.8)	69 (2.1)	122 (1.7)
Healthy weight (18.5 – 24.9)	2613 (24.8)	823 (25.0)	1790 (24.7)
Overweight (25 – 29.9)	3335 (31.6)	1020 (31.0)	2315 (31.9)
Obese (≥30)	4407 (41.8)	1379 (41.9)	3028 (41.7)
Missing	~	~	~
**Alcohol**			
Non-drinker	1268 (12.0)	360 (10.9)	908 (12.5)
Current drinker	7194 (68.2)	2308 (70.1)	4886 (67.4)
Ex-drinker	1451 (13.8)	447 (13.6)	1004 (13.8)
Missing	633 (6.0)	176 (5.4)	457 (6.3)
**Comorbidities**			
Hypertension	7928 (75.2)	2496 (75.8)	5432 (74.9)
IHD	6042 (57.3)	1919 (58.3)	4123 (56.8)
Heart Failure	6171 (58.5)	1983 (60.3)	4188 (57.7)
Arrhythmia	4495 (42.6)	1493 (45.4)	3002 (41.4)
Diabetes	3252 (30.8)	1043 (31.7)	2209 (30.5)
Peripheral Arterial Disease	946 (9.0)	326 (9.9)	6202 (8.6)
**Medication use**			
Calcium Channel Blocker	5043 (47.8)	1578 (48.0)	3465 (47.8)
Beta Blocker	6123 (58.1)	1976 (60.0)	4147 (57.2)
Thiazide diuretic	4354 (41.3)	1384 (42.1)	2970 (40.9)
Loop diuretic	7447 (70.6)	2490 (75.7)	4957 (68.3)
**eGFR category (ml/min/1.73m^2)[#]**			
≥60	5803 (55.0)	1665 (50.6)	4138 (57.0)
45-59	2668 (25.3)	864 (26.3)	1805 (24.9)
30-44	1666 (15.8)	591 (18.0)	1075 (14.8)
15-29	409 (3.9)	171 (5.2)	237 (3.3)
**Calendar time**			
2004-2006	2374 (22.5)	645 (19.6)	1729 (23.8)
2007-2009	3029 (28.7)	943 (28.7)	2086 (28.8)
2010-2014	5143 (48.8)	173 (51.8)	3440 (47.4)
**Biochemical parameters at baseline[‡]**			
Creatinine (µmol/L) (mean (SD))	99.1 (30.7)	102.7 (33.4)	97.4 (29.3)
Potassium (mmol/L) (mean (SD))	4.4 (0.7)	4.4 (0.9)	4.4 (0.6)
**Potassium (mmol/L)**			
<5.0mmol/L	8955 (84.9)	2779 (84.4)	6176 (85.1)
5.0-5.5mmol/L	1136 (10.8)	367 (11.2)	769 (10.6)

For peer review only

For peer review only - http://bmjopen.bmj.com/site/about/guidelines.xhtml

>5.5mmo/L	252 (2.4)	78 (2.4)	174 (2.4)
Missing	203 (1.9)	67 (2.0)	136 (1.9)

Data shown are column number (%), except where mean (SD) is indicated.
~ refers to negligible.
IHD-Ischaemic Heart Disease; eGFR–estimated Glomerular Filtration Rate; AA-aldosterone antagonist; ACEI/ARB-angiotensin-converting enzyme inhibitor/angiotensin receptor blocker.
[#]Calculated from most recent creatinine measurement within 12 months before the first prescription date.
[‡]Baseline biochemical parameters were the values closest to AA initiation in the preceeding year.

Testing within two weeks of AA initiation

Within two weeks post AA initiation, 31.2% of the cohort had blood testing **(Table 1).** Approximately 64% of the population had testing within two months of initiation, while 95% had blood tests within one year **(Table 2)**. We conducted a sensitivity analysis among the 5,787 individuals not admitted to hospital within 30 days before or after AA initiation (54.9% of the whole cohort). Among these patients a similar proportion (32.7%) had follow up monitoring within two weeks **(Supplementary Table 2)**. The proportion of people having six tests within a year (roughly equating to optimal guideline recommended testing within 7 days, and in months 1,2,3,6 and 12) testing was approximately 1%.

Table 2: Proportion of patients taking ACEI/ARB who initiate aldosterone antagonists in UK primary care 2004-2014 with blood tests at several timepoints after initiation of AA

7 days	14 days	1 month	2 months	3 months	6 months	1 year
1674/10613	3291/10546	5210/10431	6520/10258	7229/10145	8389/9962	9294/9799
15.8%	31.2%	49.9%	63.5%	71.3%	84.2%	94.9%

The denominator changes due to patients exiting the cohort

Factors associated with testing within two weeks of AA initiation

Women and younger patients had lower odds for blood testing within two weeks of AA initiation **(Table 3)**. Those initiating AA after 2007 had higher odds of blood testing compared to patients initiating in 2004-2006**.** Those with high baseline potassium values did not have higher odds for receiving blood testing. No substantial differences were seen when the analysis was restricted to non-hospitalised patients although the increased odds of testing among patients with reduced baseline renal function was reduced, suggesting that there were disproportionate admissions among this group **(Supplementary Table 3)**.

For peer review only - http://bmjopen.bmj.com/site/about/guidelines.xhtml

Table 3: Association between patient characteristics and follow-up monitoring within two weeks following initiation of an aldosterone antagonist

	OR (95% CI)	
	Age and sex adjusted	Fully adjusted
**Male**	ref	ref
**Female**	0.90 (0.83 – 0.98)	0.90 (0.82 – 0.98)
**Age (years)**		
<50	0.54 (0.43 – 0.67)	0.58 (0.46 – 0.73)
50-59	0.72 (0.61 – 0.84)	0.78 (0.66 – 0.91)
60-64	0.81 (0.68 – 0.96)	0.86 (0.72 – 1.03)
65-69	0.94 (0.81 – 1.09)	0.96 (0.83 – 1.12)
70-75	ref	ref
76-79	0.90 (0.83-0.98)	0.95 (0.82 – 1.10)
80+	1.05 (0.92-1.20)	0.97 (0.85 – 1.11)
**eGFR category (ml/min/1.73m^2)**		
≥60	ref	ref
45-59	1.10 (0.99 – 1.23)	1.13 (1.01 – 1.27)
30-44	1.25 (1.10 – 1.42)	1.29 (1.14 - 1.46)
15-29	1.65 (1.33 – 2.06)	1.74 (1.40 - 2.16)
**Comorbidities**		
Hypertension	1.03 (0.93 – 1.13)	0.98 (0.88 – 1.09)
Ischaemic Heart Disease	1.00 (0.92 – 1.09)	0.98 (0.89 – 1.07)
Heart Failure	1.05 (0.96 – 1.15)	1.01 (0.92 – 1.11)
Arrhythmia	1.09 (1.00 – 1.19)	1.07 (0.98 – 1.18)
Diabetes	1.06 (0.97 – 1.16)	1.05 (0.95 – 1.15)
Peripheral Arterial Disease	1.12 (0.98 – 1.27)	1.08 (0.93 – 1.24)
**Calendar time**		
2004-2006	ref	ref
2007-2009	1.22 (1.07 – 1.38)	1.21 (1.07 – 1.38)
2010-2014	1.34 (1.17 – 1.53)	1.35 (1.18 – 1.55)
**Baseline potassium**		
<5.0mmol/L	ref	ref
5.0-5.5mmol/L	1.05 (0.91 – 1.20)	0.98 (0.86 – 1.12)
>5.5mmo/L	0.98 (0.73 – 1.30)	0.90 (0.68 -1.20)

eGFR–estimated Glomerular Filtration Rate.
Fully adjusted: adjusted for age, gender, CKD stage, hypertension, heart failure, ischaemic heart disease, arrhythmias, diabetes, peripheral arterial disease, baseline potassium and calendar time. Further adjustment for lifestyle covariates made marginal difference to all results, thus these variables are not included in models shown.

For peer review only - http://bmjopen.bmj.com/site/about/guidelines.xhtml

Adverse biochemical values

Of those with follow-up monitoring within two months of AA initiation (n=6,520), 2.0% had hyperkalaemia, 2.7% a creatinine ≥220µmol/L and 13.5% a 30% increase in creatinine from baseline, on their first blood test. **(Table 4)**. Of those with creatinine ≥220µmol/L, 29 people (16%) had creatinine ≥220µmol/L at baseline. A majority of patients with hyperkalaemia discontinued AA within 30 days of the blood test **(Table 4)**. However, less than one third of those with a >30% increase in creatinine discontinued AA, compared to 43% of those with a post-initiation creatinine ≥220µmol/L. Among non-hospitalised patients, the proportions with adverse laboratory values were marginally smaller while the proportion of people discontinuing the AA with each adverse biochemical value was similar **(Supplementary Table 4)**.

Table 4: Proportion with adverse biochemical values on testing within two months of AA initiation and number subsequently discontinuing aldosterone antagonist

	Hyperkalaemia (≥6mmol/L)[‡]	Creatinine ≥220µmol/L	≥30% increase in creatinine
Number with adverse biochemical values*	128/6373 (2.0%)	177/6520 (2.7%)	877/6520 (13.5%)
Number discontinuing AA[§]	68/128 (53.1%)	76/177 (42.9%)	251/877 (28.6%)

*Serum potassium and creatinine values on first blood test within two months of AA initiation.
[‡]Missing data for 147 people (2.3%) for first follow-up potassium value.
[§]Discontinuation defined as no further prescriptions of AA after blood test plus 30 days.
AA–aldosterone antagonist.

Factors associated with adverse biochemical values

In fully adjusted logistic regression models baseline eGFR<60mls/min/1.73m^2 and potassium ≥5mmol/L were independently associated with hyperkalaemia **(Table 5)**. Women had lower odds than men for a post-initiation creatinine ≥220µmol/L, but had a higher odds of a 30% increase in creatinine. There was no consistent evidence that baseline renal function was associated with developing a 30% increase in creatinine. In sensitivity analyses of the non-hospitalised cohort, similar associations between age and sex and the occurrence of adverse biochemical values were observed **(Supplementary Table 5)**.

For peer review only - http://bmjopen.bmj.com/site/about/guidelines.xhtml

Table 5: Associations between patient characteristics and adverse biochemical values after aldosterone antagonist initiation

	OR (95% CI)		
	Hyperkalaemia[‡*] (≥6mmol/L)	Creatinine[*] ≥220µmol/L	≥30% increase in creatinine[*]
**Female**	1.04 (0.70 – 1.54)	0.34 (0.23 – 0.51)	1.47 (1.25 – 1.73)
**Age (years)**			
<50	0.15 (0.02 – 1.08)	0.96 (0.32 – 2.84)	0.34 (0.21 – 0.55)
50-59	0.29 (0.10 – 0.86)	0.80 (0.32 – 2.84)	0.45 (0.32 – 0.65)
60-64	0.60 (0.28 – 1.31)	1.17 (0.53 – 2.56)	0.69 (0.49 – 0.96)
65-69	0.83 (0.43 – 1.59)	0.36 (0.16 – 0.83)	0.64 (0.49 – 0.85)
70-75	*ref*	*ref*	*ref*
76-79	0.75 (0.43 – 1.33)	0.71 (0.42 – 1.20)	0.94 (0.75 – 1.18)
80+	0.69 (0.40 – 1.19)	0.50 (0.30 – 0.83)	1.10 (0.87 – 1.39)
**eGFR category (ml/min/1.73m^2)**			
≥60	*ref*	*ref*	*ref*
45-59	2.06 (1.26 – 3.35)	2.80 (1.22 – 6.39)	0.81 (0.66 – 0.99)
30-44	2.32 (1.31 – 4.11)	20.83 (9.73 – 44.58)	0.82 (0.67 – 1.00)
15-29	3.62 (1.60 – 8.22)	248.0 (117.2-524.7)	1.01 (0.72 – 1.40)
**Comorbidities**			
Hypertension	1.27 (0.76 – 2.14)	0.93 (0.61 – 1.41)	1.18 (0.98 – 1.41)
Ischaemic Heart Disease	0.67 (0.45 – 1.00)	0.60 (0.43 – 0.85)	0.95 (0.82 – 1.11)
Heart Failure	0.77 (0.53 – 1.14)	0.97 (0.67 – 1.41)	1.12 (0.96 – 1.31)
Arrhythmia	1.00 (0.68 – 1.46)	0.81 (0.57 – 1.15)	1.11 (0.96 – 1.28)
Diabetes	1.21 (0.83 – 1.76)	1.11 (0.80 – 1.55)	1.18 (1.01 – 1.38)
Peripheral Arterial Disease	0.82 (0.44-1.51)	1.04 (0.64- 1.68)	1.09 (0.85 – 1.40)
**Calendar time**			
2004-2006	*ref*	*ref*	*ref*
2007-2009	1.14 (0.65-2.01)	1.36 (0.84 - 2.22)	0.97 (0.80 – 1.18)
2010-2014	1.08 (0.64- 1.83)	0.93 (0.58 – 1.51)	0.95 (0.78 – 1.15)
**Baseline potassium**			
<5.0mmol/L	*ref*		
5.0-5.5mmol/L	3.59 (2.43 - 5.32)	*n/a*	*n/a*
>5.5mmo/L	3.29 (1.57 – 6.88)		

202 *Adverse serum potassium and creatinine values on first blood test within two months of AA initiation.
203 N = 6,520 except [‡]missing data for 147 people for first follow-up potassium value.
204 Only fully adjusted model is shown for clarity. Adjusted for age, gender, CKD stage, hypertension, heart failure, ischaemic
205 heart disease, arrhythmias, diabetes, peripheral arterial disease, baseline potassium (hyperkalemia model only) and
206 calendar time. Further adjustment for lifestyle covariates made marginal difference to all results and is not included.
207 eGFR–estimated Glomerular Filtration Rate.

For peer review only - http://bmjopen.bmj.com/site/about/guidelines.xhtml

DISCUSSION

In this cohort study of 10,546 users of ACEI/ARB, less than a third had follow up blood testing within two weeks of AA initiation. Two percent of the cohort developed severe hyperkalaemia on the first blood test within two months of AA initiation, and a similar proportion developed an absolute value of creatinine at which some guidelines recommend AA cessation is considered. In the same time frame, 13.5% of the cohort developed a ≥30% increase in serum creatinine from baseline.

Strengths and Weaknesses

We used a linked primary and secondary care dataset to ensure complete information on comorbid diagnoses and to allow sensitivity analyses for those without hospital admissions. Further, our data represent a general population that is unrestricted by indication for therapy, thus our results are generalisable to the vast majority of patients receiving their treatment in primary care. However, to be pragmatic we quantified testing for all patients against the NICE heart failure practical guidelines, although not all patients will have had heart failure as the indication for treatment. Even amongst those coded to have heart failure, we did not have details of patients echocardiographic findings so could not restrict to those with impaired left ventricular function, although this is the group in whom the benefits of treatment despite the risk of worsening of renal function are clearest.[22] We deliberately chose 2004 as the study start year to coincide with the roll out of the Quality and Outcomes Framework (QOF), a system of annual rewards to GPs which encouraged the standardisation of quality primary medical services. The introduction of QOF saw the incentivisation of creatinine testing amongst people with diabetes in 2004, and the establishment of a practice CKD register with the introduction of eGFR reporting in 2006.[23]

As well as examining the biochemical values of concern noted in current NICE practical guidelines for heart failure, we also assessed the incidence of a ≥30% increase in creatinine after initiation of AA. This outcome is not discussed in the practical guidelines as a parameter for consideration of the benefits of continuing AA therapy, and indeed may indicate favourable haemodynamic changes associated with improved outcomes [6]. However, worsening of renal function has been shown to have prognostic significance after initiation of ACEI/ARB treatment [24], particularly in the context of heart failure with preserved ejection fraction.[22]

A limitation is that our results capture only those who had a record of blood testing in primary care and the risk of adverse events may be different in patients who were not tested. To maximise sample size and clinical relevance we included all patients with monitoring of creatinine. Approximately 2% of these patients had missing potassium values prior to AA initiation and 2.5% after AA initiation. We did not have access to blood test results from secondary care, which could

For peer review only - http://bmjopen.bmj.com/site/about/guidelines.xhtml

have created a bias towards missing data among the sickest patients. However, restricting the analysis to individuals without any record of a hospitalisation in the 30-day period prior to and after AA initiation did not substantially change our results. The CKD-EPI equation for calculating eGFR requires an indicator for Afro-Caribbean ethnicity. Because ethnicity in our dataset was almost 50% missing, we did not use this variable in our calculations. We expect the impact of this to be small given that only 3% of the English population is of Afro-Caribbean ethnicity.[25]

Comparison with other studies

In a study of heart failure patients receiving health care through the Veterans Affairs health insurance programme in the US between 2003 and 2013, blood testing was carried out in 42% of patients within two weeks of initiation of AA.[10] Two other studies of heart failure patients carried out in North American settings found slightly higher monitoring rates for AA initiators; 53.5% within 7 days of initiation[3] and 44.6% within 10 days of initiation.[11] The contrast between our results and the results of North American research is noteworthy because it has been suggested in the past that monitoring in the UK is more frequent than in Canada.[4, 9] However, prior research on the frequency of monitoring in the UK was carried out in one geographical area and did not directly assess concordance with guideline recommended testing.[9]

Previous research examining patient characteristics associated with blood testing within 13 months of AA initiation found similar results to our study; that males, older patients and those with CKD had higher odds of receiving blood tests.[26] While diabetes was weakly associated with testing in our study (OR 1.2 in sensitivity analysis of non-hospitalised patients), the association was less pronounced than in prior research (OR 1.6).[26]

We found that 2% of those initiating an AA experienced hyperkalaemia (potassium ≥6mmol/L) on the first blood testing event within two months of AA initiation. This compares to a rate of 2% found in RALES and 5.5% in the Eplerenone Post–Acute Myocardial Infarction Heart Failure Efficacy and Survival Study (EPHESUS), both clinical trials of AAs in patients with heart failure.[1, 27] In observational studies, rates of hyperkalaemia ranging from 6% [28] to 10% [29] have been reported. Such populations are said to be more reflective of the typical user of an AA in clinical practice than in clinical trials, and thus, the rates are thought to be more realistic.[4] The disparity between our results for hyperkalaemia and those found in other observational research may have stemmed from unknown rates of adherence in this unselected population, from differences in doses used[6], or from differences in methodology. For example, we detected potassium values only on the first blood test within two months whereas prior studies examined peak potassium values within 3

For peer review only - http://bmjopen.bmj.com/site/about/guidelines.xhtml

months of initiation [28], and peak values within the individuals' entire treatment period.[29] As time since initiation increases it is increasingly likely that blood tests in primary care are performed due to an intercurrent illness or other event that may itself have contributed to hyperkalaemia or reduced renal function. Thus, we opted to look at the first blood testing event within two months to examine as much as possible a causal association between the initiation of an AA and the adverse biochemical value. In line with previous literature, we found that baseline potassium value was a significant predictor of hyperkalaemia after initiation of AA [27, 28, 30], as was reduced renal function.[26, 27, 28]

Using a threshold creatinine value as a safety indicator for renal function may not be an optimal measure given that absolute creatinine levels create an age, gender and ethnicity bias. Importantly we found that, consistent with a lower muscle mass, women had a lower odds of developing a creatinine ≥220μmol/L post AA initiation in comparison to men, but a 1.5 fold higher odds of a ≥30% increase in creatinine. The clinical implications of higher odds of ≥30% increase in creatinine are unknown but it is important to establish that there is not a discrepancy in related clinical outcomes. However, gender related differences in adverse biochemical values was not a pre-specified hypothesis so this finding must be considered hypothesis-generating. We found that approximately half of those with hyperkalaemia and creatinine ≥220μmol/L discontinued the AA within 30 days of the first post-initiation blood test which translates to an overall discontinuation rate of about 1%. This is lower than that reported in previous observational clinical research where approximately 7% of AA users required discontinuation for either hyperkalaemia or adverse renal events, although this was over the duration of therapy.[30]

Conclusions and clinical implications

We found that among patients taking an ACEI/ARB, less than one third of those initiating an AA received follow-up blood tests within two weeks. Of these, approximately 2% of patients developed potassium ≥6mmol/L or creatinine ≥220μmol/L on the first blood test within two months but 13.5% experienced a ≥30% increase in creatinine from baseline. Women had almost 50% higher odds of a ≥30% increase in creatinine than men. Baseline potassium levels >5.0mmol/L and baseline eGFR <60mls/min/1.73m^2 were independently associated with a potassium value ≥6mmol/L after AA initiation. Our results highlight the need for better adherence to monitoring guidelines, particularly for those in these high risk groups, and the importance of understanding the prognostic implications of changes in renal function after AA initiation.

For peer review only - http://bmjopen.bmj.com/site/about/guidelines.xhtml

For peer review only

DATA SHARING

No additional data available

FIGURE TITLE AND LEGEND

Figure 1: Flowchart demonstrating cohort selection

ACEI/ARB–angiotensin converting enzyme inhibitor/angiotensin receptor blocker, HES–hospital episode statistics, CPRD – Clinical Practice Research Datalink, AA-aldosterone antagonist, ESRD–end stage renal disease

For peer review only

For peer review only - http://bmjopen.bmj.com/site/about/guidelines.xhtml

REFERENCES

1 Pitt B, Zannad F, Remme WJ, et al. The Effect of Spironolactone on Morbidity and Mortality in Patients with Severe Heart Failure. *New England Journal of Medicine* 1999;**341**:709-17.
2 Williams B, MacDonald TM, Morant S, et al. Spironolactone versus placebo, bisoprolol, and doxazosin to determine the optimal treatment for drug-resistant hypertension (PATHWAY-2): a randomised, double-blind, crossover trial. *The Lancet* 2015;**386**:2059-68.
3 Allen LA, Shetterly S, Peterson PN, et al. Guideline concordance of testing for hyperkalemia and kidney dysfunction during initiation of mineralocorticoid receptor antagonist therapy in patients with heart failure. *Circulation: Heart Failure* 2013;**7**:43-50.
4 Juurlink DN, Mamdani MM, Lee DS, et al. Rates of hyperkalemia after publication of the Randomized Aldactone Evaluation Study. *New England Journal of Medicine* 2004;**351**:543-51.
5 MHRA. Drug Safety Notice. Spironolactone and renin angiotensin system drugs in heart failure: risk of potentially fatal hyperkalaemia. Available at https://www.gov.uk/drug-safety-update/spironolactone-and-renin-angiotensin-system-drugs-in-heart-failure-risk-of-potentially-fatal-hyperkalaemia 2016.
6 Pitt B, Rossignol P. The safety of mineralocorticoid receptor antagonists (MRAs) in patients with heart failure. *Expert opinion on drug safety* 2016;**15**:659-65.
7 National Institute for Health and Clinical Excellence (NICE). NICE Guideline CG108 Chronic Heart Failure. Appendix D – Practical Notes. Available at https://www.nice.org.uk/guidance/cg108/evidence/full-guideline-appendix-d-practical-notes-separate-file-136060532 2010.
8 National Institute for Health and Clinical Excellence (NICE). NICE Guideline CG127 Hypertension: Clinical management of primary hypertension in adults. 2011.
9 Wei L, Struthers AD, Fahey T, et al. Spironolactone use and renal toxicity: population based longitudinal analysis. *BMJ* 2010;**340**:c1768.
10 Chauhan V, Dev S, Pham M, et al. Facility variation and predictors of serum potassium monitoring after initiation of a mineralocorticoid receptor antagonist in patients with heart failure. *American heart journal* 2015;**170**:543-9.
11 Cooper LB, Hammill BG, Peterson ED, et al. Consistency of Laboratory Monitoring During Initiation of Mineralocorticoid Receptor Antagonist Therapy in Patients With Heart Failure. *JAMA* 2015;**314**:1973-5.
12 Herrett E, Gallagher AM, Bhaskaran K, et al. Data resource profile: clinical practice research datalink (CPRD). *International journal of epidemiology* 2015;**44**:827-36.
13 Herrett E, Thomas SL, Schoonen WM, et al. Validation and validity of diagnoses in the General Practice Research Database: a systematic review. *British journal of clinical pharmacology* 2010;**69**:4-14.
14 Herrett E, Shah AD, Boggon R, et al. Completeness and diagnostic validity of recording acute myocardial infarction events in primary care, hospital care, disease registry, and national mortality records: cohort study. *BMJ* 2013;**346**:f2350.
15 Schmidt M, Mansfield KE, Bhaskaran K, et al. Adherence to guidelines for creatinine and potassium monitoring and discontinuation following renin–angiotensin system blockade: a UK general practice-based cohort study. *BMJ Open* 2017;**7**:e012818.
16 Bhaskaran K, Douglas I, Forbes H, et al. Body-mass index and risk of 22 specific cancers: a population-based cohort study of 5· 24 million UK adults. *The Lancet* 2014;**384**:755-65.
17 Levey AS, Stevens LA, Schmid CH, et al. A New Equation to Estimate Glomerular Filtration Rate. *Annals of internal medicine* 2009;**150**:604-12.
18 Clase CM, Barzilay J, Gao P, et al. Acute change in glomerular filtration rate with inhibition of the renin-angiotensin system does not predict subsequent renal and cardiovascular outcomes. *Kidney International* 2016.
19 National Institute for Health and Clinical Excellence (NICE). NICE guideline CG182 - Chronic kidney disease in adults: assessment and management. 2014.

For peer review only - http://bmjopen.bmj.com/site/about/guidelines.xhtml

20 Mathur R, Bhaskaran K, Chaturvedi N, et al. Completeness and usability of ethnicity data in UK-based primary care and hospital databases. *Journal of public health* 2014;**36**:684-92.
21 StataCorp. Stata Statistical Software: Release 14. College Station, TX: StataCorp LP. 2015.
22 Beldhuis IE, Streng KW, Ter Maaten JM, et al. Renin-Angiotensin System Inhibition, Worsening Renal Function, and Outcome in Heart Failure Patients With Reduced and Preserved Ejection Fraction: A Meta-Analysis of Published Study Data. *Circulation Heart failure* 2017;**10**.
23 McDonald H. The epidemiology of infections among older people with diabetes mellitus and chronic kidney disease. London School of Hygiene & Tropical Medicine 2015.
24 Schmidt M, Mansfield KE, Bhaskaran K, et al. Serum creatinine elevation after renin-angiotensin system blockade and long term cardiorenal risks: cohort study. *BMJ* 2017;**356**:j791.
25 Office for National Statistics. Census: Key Statistics for local authorities in England and Wales, table number KS201EW [online]. 2012. Available from: www.ons.gov.uk/Fons/Frel/Fcensus/F2011-census/Fkey-statistics-for-local-authorities-in-england-and-wales/Frft-table-ks201ew.xls. 2012.
26 Raebel MA, McClure DL, Chan KA, et al. Laboratory evaluation of potassium and creatinine among ambulatory patients prescribed spironolactone: are we monitoring for hyperkalemia? *Annals of Pharmacotherapy* 2007;**41**:193-200.
27 Pitt B, Bakris G, Ruilope LM, et al. Serum Potassium and Clinical Outcomes in the Eplerenone Post–Acute Myocardial Infarction Heart Failure Efficacy and Survival Study (EPHESUS). *Circulation* 2008;**118**:1643-50.
28 Shah KB, Rao K, Sawyer R, et al. The adequacy of laboratory monitoring in patients treated with spironolactone for congestive heart failure. *Journal of the American College of Cardiology* 2005;**46**:845-9.
29 Svensson M, Gustafsson F, Galatius S, et al. How prevalent is hyperkalemia and renal dysfunction during treatment with spironolactone in patients with congestive heart failure? *Journal of cardiac failure* 2004;**10**:297-303.
30 Tamirisa KP, Aaronson KD, Koelling TM. Spironolactone-induced renal insufficiency and hyperkalemia in patients with heart failure. *American heart journal* 2004;**148**:971-8.

For peer review only

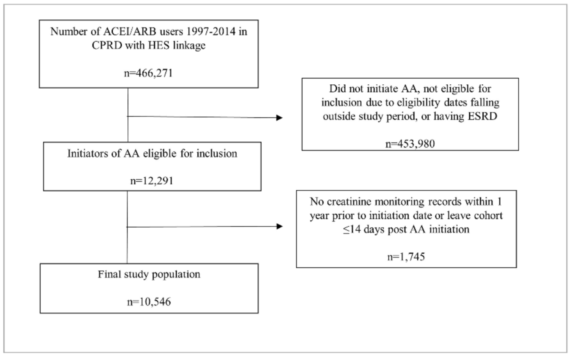

Title Figure 1: Flowchart demonstrating cohort selection
Legend ACEI/ARB–angiotensin converting enzyme inhibitor/angiotensin receptor blocker, HES–hospital episode statistics, CPRD – Clinical Practice Research Datalink, AA-aldosterone antagonist, ESRD–end stage renal disease

338x190mm (300 x 300 DPI)

view only

Supplementary Information

Table S1: Summary of clinical guideline recommendations for blood testing after initiation of AA

	NICE HF*	NICE HF Practical Guidelines Appendix D	NICE HF Practical Guidelines Appendix M	NICE Hypertension§	British National Formulary	American Guidelines∞	European Guidelines
≤7 days	*na*	x	x	*na*	x	x	x
≤1 month	*na*	x	x	x	X	x	x
≤2 month	*na*	x	x	*na*	X	x	x
≤3 month	*na*	x	x	*na*	X	x	x
≤6 month	*na*	x	x	*na*	X	x	x
≤9 month	*na*	*na*	x	*na*	X	x	x
≤12 month	*na*	x	x	*na*	X	x	x
How frequently after 1st year	*na*	6 monthly	6 monthly	*na*	6 monthly	3 monthly	4 monthly
Discontinue if K≥6mmol/L	*na*	x	x	*na*	*na*	x∞∞	x
Discontinue if creatinine ≥220µmol/L	*na*	x	x†	*na*	*na*	*na***	x‡
Discontinue if relative changes between baseline creatinine and follow up creatinine or eGFR ≥X%	*na*	*na*	*na*	*na*	*na*	*na***	*na*

* NICE HF guidelines recommend monitoring within two weeks after a medication change, but refer to Appendix D for guidance on monitoring for hyperkalaemia and renal function deterioration

† Recommends discontinuing if creatinine ≥200µmol/L

§ Recommends blood testing as required after testing within one month of AA initiation/increased diuretic use

∞ Recommends blood testing within 2-3 days of AA initiation, and again at 7 days.

∞∞ Recommends discontinuation at potassium > 5.5mEq/L

** Does not provide strict stopping/starting criteria according to serum creatinine thresholds. Suggests that risk of hyperkalaemia increases with worsening renal function and in elderly patients, or patients with low muscle mass in whom serum creatinine does not accurately reflect GFR, determination that GFR is >30ml/min/1.73m^2 is recommended.

For peer review only - http://bmjopen.bmj.com/site/about/guidelines.xhtml

‡ Recommends discontinuing if creatinine ≥310µmol/L or eGFR <20ml/min/1.73m^2
European Guidelines: "2016 ESC Guidelines for the diagnosis and treatment of acute and chronic heart failure"

American Guidelines: "2013 ACCF/AHA Guideline for the Management of Heart Failure"
NICE: National Institute for Health and Care Excellence
HF: Heart Failure
GP: General Practitioner
"x" refers to when the row condition is satisfied, "na" refers to not mentioned or not satisfied.

For peer review only

For peer review only - http://bmjopen.bmj.com/site/about/guidelines.xhtml

SI Table 2: Proportion of non-hospitalised patients taking ACEI/ARB who initiate aldosterone antagonists in UK primary care 2004-2014 with blood tests with 14 days of AA initiation

Non-hospitalised population	Monitoring ≤14 days post initiation (n., %)	No monitoring ≤14 days post initiation (n., %)
n=5787	1892 (32.7)	3897 (67.3)

Proportions for monitoring use 5787 as denominator. Non-hospitalisation refers to not having a hospitalisation in the 30 days prior to or post initiation of AA.

For peer review only

For peer review only - http://bmjopen.bmj.com/site/about/guidelines.xhtml

SI Table 3: Association between non-hospitalised patient characteristics and follow up monitoring within two weeks of initiation of an aldosterone antagonist

	OR (95% CI)	
	**Age and sex adjusted**	**Fully adjusted**
**Male**	*ref*	*ref*
**Female**	0.88 (0.78 – 0.99)	0.91 (0.80 – 1.03)
**Age (years)**		
<50	0.38 (0.28 – 0.53)	0.42 (0.30 – 0.58)
50-59	0.69 (0.56 – 0.85)	0.75 (0.60 – 0.93)
60-64	0.83 (0.66 – 1.06)	0.89 (0.70 – 1.13)
65-69	0.93 (0.76 – 1.14)	0.95 (0.77 – 1.17)
70-75	*ref*	*ref*
76-79	0.91 (0.75 – 1.11)	0.92 (0.75 – 1.12)
80+	1.13 (0.95 – 1.34)	1.11 (0.92 – 1.32)
**eGFR category (ml/min/1.73m^2)**		
≥60	*ref*	*ref*
45-59	1.00 (0.86 – 1.16)	1.02 (0.87 – 1.19)
30-44	1.10 (0.93 – 1.31)	1.10 (0.92 – 1.32)
15-29	1.13 (0.82 – 1.57)	1.15 (0.83 – 1.60)
**Comorbidities**		
Hypertension	0.98 (0.86 – 1.12)	0.96 (0.83 – 1.60)
Ischaemic Heart Disease	1.09 (0.97 – 1.23)	1.04 (0.93 – 1.17)
Heart Failure	1.17 (1.04 – 1.32)	1.11 (0.97 – 1.26)
Arrhythmia	1.16 (1.03 – 1.31)	1.10 (0.97 – 1.25)
Diabetes	1.16 (1.02 – 1.33)	1.17 (1.02 – 1.34)
Peripheral Arterial Disease	1.13 (0.93 – 1.38)	1.06 (0.87 – 1.31)
**Calendar time**		
2004-2006	*ref*	*ref*
2007-2009	1.26 (1.07 – 1.49)	1.25 (1.06 – 1.49)
2010-2014	1.36 (1.15 – 1.61)	1.35 (1.13 – 1.60)
**Baseline potassium**		
<5.0mmol/L	*ref*	*ref*
5.0-5.5mmol/L	1.05 (0.87 – 1.27)	1.01 (0.84 – 1.23)
>5.5mmo/L	0.87 (0.59 – 1.30)	0.85 (0.57 – 1.26)

eGFR–estimated Glomerular Filtration Rate.
Fully adjusted: adjusted for age, gender, CKD stage, hypertension, heart failure, ischaemic heart disease, arrhythmias, diabetes, peripheral arterial disease, baseline potassium and calendar time. Further adjustment for lifestyle covariates and socioeconomic status made marginal difference to all results, thus these variables are not included in models shown.

For peer review only - http://bmjopen.bmj.com/site/about/guidelines.xhtml

SI Table 4: Proportion of non-hospitalised patients with adverse biochemical values on testing within 2 months of AA initiation and number subsequently discontinuing aldosterone antagonist

	**Hyperkalaemia (>=6mmol/L)[‡]**	**Creatinine (≥220µmol/L)**	**≥30% Change in Creatinine**
Number with adverse biochemical values (n,%)*	60/3698 (1.6%)	75/3757 (2.0%)	374/3757 (10.0%)
Number discontinuing AA (n, %)[∞]	30/60 (50.0%)	29/75 (38.7%)	109/374 (29.1%)

*serum potassium and creatinine values on first blood test within two months of AA initiation.
[‡]Missing data for 59 people for first follow-up potassium value.
[§]Discontinuation defined as no further prescriptions of AA after blood test plus 30 days.
AA–aldosterone antagonist.

For peer review only

For peer review only - http://bmjopen.bmj.com/site/about/guidelines.xhtml

SI Table 5: Associations between non-hospitalised patient characteristics and adverse biochemical values after aldosterone antagonist initiation

	OR (95% CI)		
	**Hyperkalaemia* (>=6mmol/L)**	**Creatinine* (≥220µmol/L)**	**≥30% Change in Creatinine**
**Male**	*ref*	*ref*	*ref*
**Female**	1.53 (0.93 – 2.51)	0.78 (0.50 – 1.23)	1.52 (1.22 – 1.90)
**Age (years)**			
<50	-	0.80 (0.22 – 2.87)	0.37 (0.16 – 0.85)
50-59	0.47 (0.15 – 1.45)	0.45 (0.16 – 1.30)	0.67 (0.40 – 1.13)
60-64	0.57 (0.18 – 1.79)	0.67 (0.23 – 1.94)	0.79 (0.47 – 1.30)
65-69	0.41 (0.13 – 1.28)	0.20 (0.05 – 0.81)	0.81 (0.52 – 1.26)
70-75	*ref*	*ref*	*ref*
76-79	0.93 (0.44 - 1.97)	1.34 (0.65 – 2.78)	1.05 (0.73 – 1.51)
80+	0.82 (0.41 - 1.64)	1.22 (0.63 – 2.37)	1.25 (0.87 – 1.80)
**eGFR category (ml/min/1.73m^2)**			
≥60	*ref*		
45-59	3.36 (1.40 – 8.05)	5.02 (1.24 – 20.24)	0.84 (0.64 – 1.10)
30-44	6.01 (2.42 – 14.89)	47.79 (13.70 – 166.76)	1.11 (0.81 – 1.53)
15-29	8.69 (2.51 – 30.10)	875.60 (240.46 – 3188.32)	1.23 (0.75 – 2.04)
**Comorbidities**			
Hypertension	1.11 (0.51 – 2.37)	1.12 (0.60 -2.08)	1.30 (0.98 - 1.73)
Ischaemic Heart Disease	0.52 (0.28 – 0.96)	0.59 (0.33 – 1.07)	1.03 (0.84 -1.27)
Heart Failure	0.72 (0.42 – 1.22)	0.98 (0.55 -1.73)	0.92 (0.73 - 1.15)
Arrhythmia	0.93 (0.54 – 1.61)	0.57 (032 – 1.01)	1.03 (0.82 -1.29)
Diabetes	0.96 (0.53 – 1.74)	1.10 (0.61 – 1.98)	0.97 (0.76 – 1.24)
Peripheral Arterial Disease	1.05 (0.38 – 2.87)	0.55 (0.21 – 1.40)	0.89 (0.59 - 1.34)
**Calendar time**			
2004-2006			
2007-2009	0.94 (0.42 – 2.07)	1.19 (0.60 – 2.38)	1.01 (0.75 – 1.35)
2010-2014	0.91 (0.46 – 1.77)	0.76 (0.40 – 1.47)	0.96 (0.72 – 1.27)
**Baseline potassium**			
<5.0mmol/L			
5.0-5.5mmol/L	2.52 (1.31 – 4.83)	*n/a*	*n/a*
>5.5mmo/L	5.26 (1.87 – 14.81)		

*Adverse serum potassium and creatinine values on first blood test within two months of AA initiation.
N = 3,757 except missing data for 59 people for first follow-up potassium value.
Only fully adjusted model is shown for clarity. Adjusted for age, gender, CKD stage, hypertension, heart failure, ischaemic heart disease, arrhythmias, diabetes, peripheral arterial disease, baseline potassium (hyperkalemia model only) and calendar time. Further adjustment for lifestyle covariates and socioeconomic status made marginal difference to all results and is not presented.
eGFR–estimated Glomerular Filtration Rate.

For peer review only

STROBE Statement—checklist of items that should be included in reports of observational studies

	Item No	Recommendation	Page
**Title and abstract**	1	(*a*) Indicate the study's design with a commonly used term in the title or the abstract	1
		(*b*) Provide in the abstract an informative and balanced summary of what was done and what was found	3
**Introduction**			
Background/rationale	2	Explain the scientific background and rationale for the investigation being reported	5
Objectives	3	State specific objectives, including any prespecified hypotheses	5
**Methods**			
Study design	4	Present key elements of study design early in the paper	6
Setting	5	Describe the setting, locations, and relevant dates, including periods of recruitment, exposure, follow-up, and data collection	6+7
Participants	6	(*a*) *Cohort study*—Give the eligibility criteria, and the sources and methods of selection of participants. Describe methods of follow-up<br>*Case-control study*—Give the eligibility criteria, and the sources and methods of case ascertainment and control selection. Give the rationale for the choice of cases and controls<br>*Cross-sectional study*—Give the eligibility criteria, and the sources and methods of selection of participants	6
		(*b*) *Cohort study*—For matched studies, give matching criteria and number of exposed and unexposed<br>*Case-control study*—For matched studies, give matching criteria and the number of controls per case	na
Variables	7	Clearly define all outcomes, exposures, predictors, potential confounders, and effect modifiers. Give diagnostic criteria, if applicable	7+6
Data sources/ measurement	8*	For each variable of interest, give sources of data and details of methods of assessment (measurement). Describe comparability of assessment methods if there is more than one group	6
Bias	9	Describe any efforts to address potential sources of bias	8
Study size	10	Explain how the study size was arrived at	10 (flowchart)
Quantitative variables	11	Explain how quantitative variables were handled in the analyses. If applicable, describe which groupings were chosen and why	6
Statistical methods	12	(*a*) Describe all statistical methods, including those used to control for confounding	8
		(*b*) Describe any methods used to examine subgroups and interactions	8
		(*c*) Explain how missing data were addressed	6
		(*d*) *Cohort study*—If applicable, explain how loss to follow-up was addressed<br>*Case-control study*—If applicable, explain how matching of cases and controls was addressed<br>*Cross-sectional study*—If applicable, describe analytical methods	

For peer review only

For peer review only - http://bmjopen.bmj.com/site/about/guidelines.xhtml

taking account of sampling strategy	
(*e*) Describe any sensitivity analyses	8

Results			Page
Participants	13*	(a) Report numbers of individuals at each stage of study—eg numbers potentially eligible, examined for eligibility, confirmed eligible, included in the study, completing follow-up, and analysed	Flowchart pg 10
		(b) Give reasons for non-participation at each stage	Flowchart pg 10
		(c) Consider use of a flow diagram	Flowchart pg 10
Descriptive data	14*	(a) Give characteristics of study participants (eg demographic, clinical, social) and information on exposures and potential confounders	Table1
		(b) Indicate number of participants with missing data for each variable of interest	Table 1 pg 11
		(c) *Cohort study*—Summarise follow-up time (eg, average and total amount)	Na
Outcome data	15*	*Cohort study*—Report numbers of outcome events or summary measures over time	Table 2+4
		Case-control study—Report numbers in each exposure category, or summary measures of exposure	
		Cross-sectional study—Report numbers of outcome events or summary measures	
Main results	16	(*a*) Give unadjusted estimates and, if applicable, confounder-adjusted estimates and their precision (eg, 95% confidence interval). Make clear which confounders were adjusted for and why they were included	Tables 3 +5
		(*b*) Report category boundaries when continuous variables were categorized	Tables 3+5
		(*c*) If relevant, consider translating estimates of relative risk into absolute risk for a meaningful time period	Na
Other analyses	17	Report other analyses done—eg analyses of subgroups and interactions, and sensitivity analyses	12+15
**Discussion**			
Key results	18	Summarise key results with reference to study objectives	16
Limitations	19	Discuss limitations of the study, taking into account sources of potential bias or imprecision. Discuss both direction and magnitude of any potential bias	16
Interpretation	20	Give a cautious overall interpretation of results considering objectives, limitations, multiplicity of analyses, results from similar studies, and other relevant evidence	16-18
Generalisability	21	Discuss the generalisability (external validity) of the study results	16
**Other information**			
Funding	22	Give the source of funding and the role of the funders for the present study and, if applicable, for the original study on which the present article is based	2

*Give information separately for cases and controls in case-control studies and, if applicable, for exposed and unexposed groups in cohort and cross-sectional studies.

Note: An Explanation and Elaboration article discusses each checklist item and gives methodological background and published examples of transparent reporting. The STROBE checklist is best used in conjunction with this article (freely available on the Web sites of PLoS Medicine at http://www.plosmedicine.org/, Annals of Internal Medicine at

For peer review only

http://www.annals.org/, and Epidemiology at http://www.epidem.com/). Information on the STROBE Initiative is available at www.strobe-statement.org.

For peer review only

For peer review only - http://bmjopen.bmj.com/site/about/guidelines.xhtml

BMJ Open

Laboratory monitoring after initiation of aldosterone antagonist therapy in users of renin-angiotensin system blockers: A UK primary care cohort study

Journal:	*BMJ Open*
Manuscript ID	bmjopen-2017-018153.R2
Article Type:	Research
Date Submitted by the Author:	09-Oct-2017
Complete List of Authors:	Sinnott, Sarah-Jo; London School of Hygiene and Tropical Medicine, Non-communicable disease epidemiology<br>Mansfield, Kathryn; London School of Hygiene and Tropical Medicine, Non-communicable disease epidemiology<br>Schmidt, Morten; London School of Hygiene and Tropical Medicine, Non-communicable disease epidemiology; Aarhus Universitet, Dept of Clinical Epidemiology<br>Bhaskaran, Krishnan; London School of Hygiene and Tropical Medicine, Non-communicable disease epidemiology<br>Smeeth, Liam; London School of Hygiene and Tropical Medicine, Non-communicable disease epidemiology<br>Nitsch, Dorothea; London School of Hygiene and Tropical Medicine, Non-communicable disease epidemiology<br>Tomlinson, Laurie; London School of Hygiene and Tropical Medicine, Non-communicable disease epidemiology
**Primary Subject Heading**:	Cardiovascular medicine
Secondary Subject Heading:	Cardiovascular medicine, Epidemiology, Health services research, Pharmacology and therapeutics
Keywords:	drug monitoring, Heart failure < CARDIOLOGY, Hypertension < CARDIOLOGY, electronic health records, health care delivery

SCHOLARONE™
Manuscripts

Laboratory monitoring after initiation of aldosterone antagonist therapy in users of renin-angiotensin system blockers: A UK primary care cohort study

Authors:

Sarah-Jo Sinnott[1], Kathryn E Mansfield[1], Morten Schmidt[1,2,3] Krishnan Bhaskaran[1], Liam Smeeth[1], Dorothea Nitsch[1] Laurie A Tomlinson[1]

1. Department of non-communicable disease epidemiology, London School of Hygiene and Tropical Medicine, Keppel St, London, WC1E7HT
2. Department of Clinical Epidemiology, Aarhus University Hospital, Denmark
3. Department of Cardiology, Regional Hospital West Jutland, Herning, Denmark

Corresponding Author:

Sarah-Jo Sinnott
Department of non-communicable disease epidemiology, London School of Hygiene & Tropical Medicine, Keppel St, London, WC1E7HT
Email: sarah-jo.sinnott@lshtm.ac.uk
Phone 02072994821

Word count

~3400

Abstract count

212

Tables and figures

1 Figure

5 Tables

Keywords

Drug monitoring, heart failure, hypertension, electronic medical records, health care delivery.

For peer review only - http://bmjopen.bmj.com/site/about/guidelines.xhtml

For peer review only

COPYRIGHT/LICENCSE FOR PUBLICATION

"The Corresponding Author has the right to grant on behalf of all authors and does grant on behalf of all authors, an exclusive licence (or non-exclusive for government employees) on a worldwide basis to the BMJ Publishing Group Ltd to permit this article (if accepted) to be published in BMJ editions and any other BMJPGL products and sublicenses such use and exploit all subsidiary rights, as set out in our licence."

CONTRIBUTORSHIP

LAT had the idea for the study and acquired data permissions. SJS, MS, KEM, DN and LAT designed the study. SJS, KEM and MS managed the data and established the cohort. SJS did the analyses. All authors (SJS, KEM, MS, KB, LS, DN, LAT) participated in the discussion and interpretation of the results. SJS organised the writing and wrote the initial drafts. All authors (SJS, KEM, MS, KB, LS, DN, LAT) critically revised the manuscript for intellectual content and approved the final version. SJS is the guarantor.

TRANSPARENCY

SJS affirms that the manuscript is an honest, accurate, and transparent account of the study being reported; that no important aspects of the study have been omitted; and that any discrepancies from the study as planned have been explained.

ETHICS

The protocol for this study was approved by London School of Hygiene and Tropical Medicine Ethics Committee (No. 6536) and the Independent Scientific Advisory Committee (ISAC) for Medicines and Healthcare Products Regulatory Agency (No. 16_025A).

FUNDING

SJS is supported by a Wellcome Trust Sir Henry Wellcome Fellowship [107340/Z/15/Z]. LS is supported by a Wellcome Trust Senior Research Fellowship in Clinical Science [098504/Z/12/Z]. LAT is supported by a Wellcome Trust Intermediate Clinical Fellowship to LAT [101143/Z/13/Z] which also funded KEM. KB is supported by a Sir Henry Dale Fellowship jointly funded by the Wellcome Trust and the Royal Society [107731/Z/15/Z]. MS was supported by the A.P. Møller Foundation for the Advancement of Medical Science, Snedkermester Sophus Jacobsen & Hustru Astrid Jacobsens Fond, and Christian og Ottilia Brorsons Rejselegat for yngre videnskabsmænd og–kvinder.

COMEPETING INTERESTS

All authors have completed the ICMJE uniform disclosure form at www.icmje.org/coi_disclosure.pdf and declare: SJS, KEM, MS, DN and LAT nothing to declare; LS reports grants from Wellcome Trust and British Heart Foundation during the conduct of the study, grants from Wellcome Trust, Medical Research Council, National Institute for Health Research and the European Union outside the submitted work, personal fees from GSK for advisory work unrelated to the submitted work, grant funding from GSK for academic research unrelated to the submitted work, acts as an unpaid steering committee chair for AstraZeneca for a randomised trial unrelated to the submitted work, and is a trustee of the British Heart Foundation. KB declares grants from the Wellcome Trust, Royal Society, MRC, NIHR and BHF outside the submitted work.

For peer review only - http://bmjopen.bmj.com/site/about/guidelines.xhtml

ABSTRACT

Objective

To determine the frequency of biochemical monitoring after initiation of aldosterone antagonists in patients also using angiotensin-converting enzyme inhibitor/angiotensin receptor blockers (ACEI/ARB).

Setting

UK primary care.

Participants

ACEI/ARB users who initiated an AA between 2004-2014.

Outcomes

We calculated the proportions with: 1) biochemical monitoring ≤2 weeks post AA initiation; 2) adverse biochemical values ≤2 months (potassium ≥6mmol/L; creatinine ≥220µmol/L; ≥30% increase in creatinine from baseline); 3) discontinuers of AA in those with an adverse biochemical value. We used logistic regression to study patient characteristics associated with testing and adverse biochemical values.

Results

In 10,546 initiators of AA, 3,291 (31.2%) had a record of biochemical monitoring ≤2 weeks post initiation. A total of 2.0% and 2.7% of those with follow-up testing within 2 months of initiation experienced potassium ≥6mmol/L and creatinine ≥220µmol/L respectively, while 13.5% had a ≥30% increase in creatinine. Baseline potassium (OR 3.59, 95% CI 2.43-5.32 for 5.0-5.5mmol/L compared to <5.0mmol/L) and eGFR 45-59mls/min/1.73m^2 (OR 2.06, 95% CI 1.26–3.35 compared to ≥60mls/min/1.73m^2) were independently predictive of potassium ≥6mmol/L. Women and people with diabetes had higher odds of ≥30% increase in creatinine.

Conclusion

Less than one third of patients taking ACEI/ARB had biochemical monitoring within 2 weeks of initiating aldosterone antagonists. Higher levels of monitoring may reduce adverse biochemical events.

For peer review only

STRENGTHS AND LIMITATIONS OF THE STUDY

- This is a population cohort study, based on electronic health records from UK primary care, examining whether users of renin-angiotensin system blockade who commence aldosterone antagonists (AA) have appropriate biochemical monitoring after initiation of AA.
- The population was not restricted by indication for therapy.
- Those who were hospitalised prior to or immediately after initiating an AA may have had missing data for test results in primary care data. In a sensitivity analysis, we used primary care data linked to hospital data to assess monitoring in a population that was not hospitalised. We found similar rates of monitoring and adverse events compared to the main analysis.

For peer review only

INTRODUCTION

Angiotensin-converting enzyme inhibitors or angiotensin receptor blockers (ACEI/ARB) and aldosterone antagonists (AA), such as spironolactone and eplenerone, are frequently used in combination. They provide reductions in morbidity and mortality for patients with heart failure [1] and reductions in blood pressure for patients with resistant hypertension.[2] However, users of these drugs are at risk of acute kidney injury [3], hyperkalaemia, and hyperkalaemia-associated mortality.[4]

The occurrence of adverse events associated with combined ACEI/ARB and AA use was highlighted in early 2016 in the UK with the publication of a drug safety notice from the Medicines and Healthcare products Regulatory Agency (MHRA). It reported on the increasing incidence of life-threatening hyperkalaemic adverse events in patients prescribed ACEI/ARB and spironolactone.[5]

To help avoid adverse events after initiation of AA, biochemical parameters should be monitored.[6] At present, the National Institute of Health and Care Excellence (NICE) practical guidelines for heart failure recommend testing for potassium, creatinine, and estimated Glomerular Filtration Rate (eGFR) after one week, and at one, two, three, and six months, and six monthly thereafter, following initiation of an AA in heart failure.[7] These guidelines recommend stopping the AA if potassium ≥6mmol/L and if creatinine ≥220µmol/L. NICE guidelines for hypertension state that testing for sodium, potassium and renal function should occur within one month after initiation of AA, and as required thereafter.[8]

It is not known how well these guidelines are adhered to in the UK. Previous evidence on blood testing during AA treatment in the UK is historic, restricted to one geographical region, and did not specifically assess adherence to guideline recommended blood testing.[9] Data from North America suggest that recommended blood testing occurs in less than 50% of patients.[3, 10, 11] Poor monitoring of patients taking these drug combinations, as well as increasing use among patients at high risk of adverse outcomes, may help to explain the increased occurrence of hyperkalaemic events as reported by the MHRA.

Therefore, among a large, recent cohort of users of ACEI/ARB who initiated an AA, we sought to examine patterns of blood testing, and the occurrence of hyperkalaemia and renal impairment. Our aims were to determine: 1) the proportion of patients initiating an AA who had testing within two weeks of initiation; 2) the patient characteristics associated with testing; 3) the proportion of patients who had adverse biochemical values after initiation of AA, and the proportion that then discontinued the AA and; 4) the patient characteristics associated with adverse biochemical values.

For peer review only - http://bmjopen.bmj.com/site/about/guidelines.xhtml

METHODS

Data

The Clinical Practice Research Database (CPRD-GOLD) is a nationally representative repository of de-identified electronic medical records from primary care in the UK. It holds data on demographics, health related behaviours, test results, diagnoses, referrals and prescriptions for more than 11 million people with research-quality data.[12] It is one of the largest databases of longitudinal medical records from primary care globally and has been extensively validated.[12, 13]

For this study, we used CPRD-GOLD data linked to Hospital Episodes Statistics (HES). This linkage is possible for 60% of English practices contributing to the CPRD-GOLD database. The HES database provides data on the primary diagnosis for a hospital admission, as well as other diagnoses and procedures carried out during that admission. The linkage thus provides a more complete picture of comorbidities, improves the accuracy of timing and in this study allowed the conduct of sensitivity analyses in those without hospital admissions.[14]

Population

We identified a cohort of HES-linked CPRD patients aged ≥18 years, who initiated ACEI/ARB treatment between April 1, 1997 and March 31, 2014.[15] We identified continuous courses of ACEI/ARB therapy by allowing for a 90-day gap between the end date of one prescription and the start of the next consecutive prescription (to allow for stockpiling and medications prescribed in secondary care). Among this cohort, we identified people who subsequently became new users of AA in the period 2004-2014. We chose 2004 as the study start year to coincide with the roll out of the Quality and Outcomes Framework (QOF), a system of remuneration to GPs which encouraged the standardisation of quality primary medical services. The introduction of QOF resulted in much higher levels and recording of renal function testing due to the incentivisation of creatinine testing amongst people with diabetes in 2004, and the establishment of a practice CKD register with the introduction of eGFR reporting in 2006.[16]

New use of AA was defined as no use of AA in the year prior to first prescription. We then restricted the population to those with a record for creatinine monitoring in the year prior to AA initiation. We assumed that those with creatinine test results prior to and after AA initiation were also tested for potassium. If values for potassium were not present they were treated as missing. This approach avoided exclusion of patients whose blood sample may have been haemolysed, resulting in potassium value not reported. Patients with READ codes for end-stage renal disease and eGFR values corresponding with chronic kidney disease stage 5 prior to cohort entry were excluded.

For peer review only - http://bmjopen.bmj.com/site/about/guidelines.xhtml

Patients were eligible for follow-up until the earliest of; death, transfer out of practice, last data collection or end of the study (March 2014).

Covariates

We obtained information for all patients on age, gender, calendar time of AA initiation (2004-2006, 2007-2009, 2010-2014), and lifestyle factors. The closest records to AA initiation date were used for determining smoking, alcohol and body mass index status using existing algorithms.[17] We extracted data on cardiovascular comorbidities and diabetes using data from both CPRD and HES. We calculated baseline eGFR using the most recent creatinine value from CPRD data prior to AA initiation and the Chronic Kidney Disease Epidemiology Collaboration (CKD-EPI) equation.[18] CPRD prescribing data were used to extract data on baseline medication use. Baseline potassium values were categorised as: <5mmol/L, 5–5.5mmol/L and >5.5mmol/L.

Outcomes and Statistical Analyses

Several guidelines recommend time periods for blood testing after initiation of an AA **(Supplementary Table 1)**. We assessed whether monitoring occurs in line with current NICE recommendations, with some modification. NICE practical guidelines for heart failure recommend blood testing within one week after AA initiation.[7] However, we calculated the proportion of AA initiators who had blood testing within two weeks of AA initiation to accommodate the practical challenges of immediate follow-up testing faced in clinical practice. In additional analyses, we also calculated the proportion of people who had testing 1) within seven days post AA initiation, 2) within one month, two months, 3 months, 6 months and 12 months post AA initiation and 3) on all recommended monitoring occasions. Guidelines offer a framework for clinicians on how to treat and manage patients but adherence to guidelines is not obligatory and deviations may just reflect individualised care. Nonetheless, using clinical guidelines to frame this analysis provides a series of time points against which we can quantify frequency of monitoring for patients using combinations of AA and ACEI/ARB.

We used thresholds set out by the NICE practical guidelines for heart failure to calculate the proportion of patients who had an adverse biochemical value on their first blood test within two months of initiation.[7] Hyperkalaemia was a potassium of ≥6mmol/L and an adverse creatinine value indicating renal dysfunction was defined as creatinine ≥220µmol/L. We chose two months to accord with monitoring periods in previous clinical trials[19], and also because at the outset we expected that a majority of patients would have testing within this timeframe. We then calculated the proportion of those with adverse biochemical values who discontinued the AA. We used a

For peer review only - http://bmjopen.bmj.com/site/about/guidelines.xhtml

conservative definition of discontinuation, to prevent misclassification of people who had blood tests at the beginning of a median length prescription (28 days). Therefore, we classified discontinuation as no further prescription of an AA beyond 30 days after the first post-initiation blood test, i.e., when the end date of the course of AA therapy occurred before the first blood test date plus 30 days.

Some practical guidelines for initiation of AA refer to an absolute level of creatinine to indicate renal dysfunction of concern and when stopping the AA should be considered (**Supplementary Table 1)**. However, absolute values of creatinine reflect substantially different levels of eGFR depending on the age, gender and ethnicity of the patient. A proportional change in creatinine or eGFR is recommended by NICE guidelines to indicate significant change in renal function for patients who initiate ACEI/ARB.[20] This measure is clinically familiar and more closely indicates changes in renal function that may be associated with a new drug. Therefore, we also calculated the number of people who experienced a 30% relative increase in creatinine from baseline as an adverse biochemical finding. We used the most recent values for creatinine within one year prior to AA initiation and creatinine values on the first blood test within two months post AA initiation to calculate the relative change.

To assess patient level characteristics associated with testing within two weeks (versus not having testing within two weeks) we used logistic regression with robust standard errors to adjust for correlations between patients within practices.[10] The crude model adjusted for age and gender only, while the fully adjusted model adjusted for age, gender, eGFR category, cardiovascular comorbidities, diabetes, baseline potassium and calendar time. We did not include ethnicity in fully adjusted models due to missing data for approximately 50% of the population.[21] We also used logistic regression to assess patient characteristics associated with an adverse biochemical value (versus not having an adverse biochemical value), with adjustments for the same variables as in the prior model.

In a sensitivity analysis, we assessed the proportion of patients receiving blood tests among patients who were not hospitalised in the 30-day period prior to or after AA initiation. Data on laboratory tests are not available in the HES database, and test results may not always be sent from the hospital to the GP practice. Therefore, this analysis excluded patients who had blood tests as inpatients, which would contribute to an apparently low observed level of testing in primary care.

We used Stata version 14 for all analyses.[22]

For peer review only - http://bmjopen.bmj.com/site/about/guidelines.xhtml

The protocol for this study was approved by London School of Hygiene and Tropical Medicine Ethics Committee (No. 6536) and the Independent Scientific Advisory Committee (ISAC) for Medicines and Healthcare Products Regulatory Agency (No. 16_025A).

For peer review only - http://bmjopen.bmj.com/site/about/guidelines.xhtml

RESULTS

From 466,271 continuous users of ACEI/ARB between 1997 and March 2014, 12,291 people initiated an AA between 2004-2014 and were eligible for inclusion. After further exclusions, 10,546 were ultimately included in the cohort. **(Figure 1)**. The population was 41% female and had a mean age of 71.8 years (SD 12.9) **(Table 1)**. Mean baseline serum potassium was 4.4 mmol/L (SD 0.7). Approximately one fifth of the population had eGFR <30mls/min/1.73m^2 **(Table 1).** Spironolactone was the drug commenced for 9,917/10,546 (94%) of those initiating an AA, with the remainder initiating eplenerone.

Insert Figure 1 here

For peer review only

For peer review only - http://bmjopen.bmj.com/site/about/guidelines.xhtml

Table 1: Characteristics of patients taking ACEI/ARB who initiate aldosterone antagonists in UK primary care 2004-2014, by monitoring groups

	Population	Monitoring ≤2 weeks	No monitoring ≤2 weeks
**Total number**	10546 (100)	3291 (100)	7255 (100)
**Female sex**	4348 (41.2)	1326 (40.3)	3022 (41.6)
**Age (years)**			
<50	656 (6.2)	138 (4.2)	518 (7.1)
50-59	1188 (11.3)	314 (9.5)	874 (12.1)
60-64	957 (9.1)	275 (8.4)	682 (9.4)
65-69	1227 (11.6)	391 (11.9)	836 (11.5)
70-74	1513 (14.4)	502 (15.3)	1011 (13.9)
75-79	1708 (16.2)	552 (16.8)	1156 (15.9)
≥80+	3297 (31.3)	1119 (34.0)	2178 (30.0)
**Ethnicity**			
White	4687 (44.4)	1476 (44.9)	3211 (44.3)
South Asian	119 (1.1)	33 (1.0)	86 (1.2)
Black	101 (1.0)	23 (0.7)	78 (1.1)
Mixed/Other	58 (0.6)	11 (0.3)	47 (0.7)
Missing/not stated	5581 (52.9)	1748 (53.1)	3833 (52.8)
**Smoking**			
Non-smoker	2693 (25.5)	835 (25.4)	1858 (25.6)
Current smoker	1505 (14.3)	405 (12.3)	1100 (15.2)
Ex-smoker	6333 (60.1)	2047 (62.2)	4286 (59.1)
Missing	15 (0.1)	~	11 (0.2)
**Body Mass Index (kg/m^2)**			
Underweight (<18.5)	191 (1.8)	69 (2.1)	122 (1.7)
Healthy weight (18.5 – 24.9)	2613 (24.8)	823 (25.0)	1790 (24.7)
Overweight (25 – 29.9)	3335 (31.6)	1020 (31.0)	2315 (31.9)
Obese (≥30)	4407 (41.8)	1379 (41.9)	3028 (41.7)
Missing	~	~	~
**Alcohol**			
Non-drinker	1268 (12.0)	360 (10.9)	908 (12.5)
Current drinker	7194 (68.2)	2308 (70.1)	4886 (67.4)
Ex-drinker	1451 (13.8)	447 (13.6)	1004 (13.8)
Missing	633 (6.0)	176 (5.4)	457 (6.3)
**Comorbidities**			
Hypertension	7928 (75.2)	2496 (75.8)	5432 (74.9)
IHD	6042 (57.3)	1919 (58.3)	4123 (56.8)
Heart Failure	6171 (58.5)	1983 (60.3)	4188 (57.7)
Arrhythmia	4495 (42.6)	1493 (45.4)	3002 (41.4)
Diabetes	3252 (30.8)	1043 (31.7)	2209 (30.5)
Peripheral Arterial Disease	946 (9.0)	326 (9.9)	6202 (8.6)
**Medication use**			
Calcium Channel Blocker	5043 (47.8)	1578 (48.0)	3465 (47.8)
Beta Blocker	6123 (58.1)	1976 (60.0)	4147 (57.2)
Thiazide diuretic	4354 (41.3)	1384 (42.1)	2970 (40.9)
Loop diuretic	7447 (70.6)	2490 (75.7)	4957 (68.3)
**eGFR category (ml/min/1.73m^2)[#]**			
≥60	5803 (55.0)	1665 (50.6)	4138 (57.0)
45-59	2668 (25.3)	864 (26.3)	1805 (24.9)
30-44	1666 (15.8)	591 (18.0)	1075 (14.8)
15-29	409 (3.9)	171 (5.2)	237 (3.3)
**Calendar time**			
2004-2006	2374 (22.5)	645 (19.6)	1729 (23.8)
2007-2009	3029 (28.7)	943 (28.7)	2086 (28.8)
2010-2014	5143 (48.8)	173 (51.8)	3440 (47.4)
**Biochemical parameters at baseline[‡]**			
Creatinine (µmol/L) (mean (SD))	99.1 (30.7)	102.7 (33.4)	97.4 (29.3)
Potassium (mmol/L) (mean (SD))	4.4 (0.7)	4.4 (0.9)	4.4 (0.6)
**Potassium (mmol/L)**			
<5.0mmol/L	8955 (84.9)	2779 (84.4)	6176 (85.1)
5.0-5.5mmol/L	1136 (10.8)	367 (11.2)	769 (10.6)

For peer review only - http://bmjopen.bmj.com/site/about/guidelines.xhtml

For peer review only

>5.5mmo/L	252 (2.4)	78 (2.4)	174 (2.4)
Missing	203 (1.9)	67 (2.0)	136 (1.9)

Data shown are column number (%), except where mean (SD) is indicated.
~ refers to negligible.
IHD-Ischaemic Heart Disease; eGFR–estimated Glomerular Filtration Rate; AA-aldosterone antagonist; ACEI/ARB-angiotensin-converting enzyme inhibitor/angiotensin receptor blocker.
[#]Calculated from most recent creatinine measurement within 12 months before the first prescription date.
[‡]Baseline biochemical parameters were the values closest to AA initiation in the preceding year.

Testing within two weeks of AA initiation

Within two weeks post AA initiation, 31.2% of the cohort had blood testing **(Table 1).** Approximately 64% of the population had testing within two months of initiation, while 95% had blood tests within one year **(Table 2)**. We conducted a sensitivity analysis among the 5,787 individuals not admitted to hospital within 30 days before or after AA initiation (54.9% of the whole cohort). Among these patients a similar proportion (32.7%) had follow-up monitoring within two weeks **(Supplementary Table 2)**. The proportion of people having six tests within a year (roughly equating to optimal guideline recommended testing within 7 days, and in months 1,2,3,6 and 12) testing was approximately 1%.

Table 2: Proportion of patients taking ACEI/ARB who initiate aldosterone antagonists in UK primary care 2004-2014 with blood tests at several time points after initiation of AA

7 days	14 days	1 month	2 months	3 months	6 months	1 year
1674/10613	3291/10546	5210/10431	6520/10258	7229/10145	8389/9962	9294/9799
15.8%	31.2%	49.9%	63.5%	71.3%	84.2%	94.9%

The denominator changes due to patients exiting the cohort

Factors associated with testing within two weeks of AA initiation

Women and younger patients had lower odds for blood testing within two weeks of AA initiation **(Table 3)**. Those initiating AA after 2007 had higher odds of blood testing compared to patients initiating in 2004-2006**.** Those with high baseline potassium values did not have higher odds for receiving blood testing. No substantial differences were seen when the analysis was restricted to non-hospitalised patients although the increased odds of testing among patients with reduced baseline renal function was reduced, suggesting that there were disproportionate admissions among this group **(Supplementary Table 3)**.

For peer review only

For peer review only - http://bmjopen.bmj.com/site/about/guidelines.xhtml

Table 3: Association between patient characteristics and follow-up monitoring within two weeks following initiation of an aldosterone antagonist

	OR (95% CI)	
	**Age and sex adjusted**	**Fully adjusted**
**Male**	ref	ref
**Female**	0.90 (0.83 – 0.98)	0.90 (0.82 – 0.98)
**Age (years)**		
<50	0.54 (0.43 – 0.67)	0.58 (0.46 – 0.73)
50-59	0.72 (0.61 – 0.84)	0.78 (0.66 – 0.91)
60-64	0.81 (0.68 – 0.96)	0.86 (0.72 – 1.03)
65-69	0.94 (0.81 – 1.09)	0.96 (0.83 – 1.12)
70-75	ref	ref
76-79	0.90 (0.83-0.98)	0.95 (0.82 – 1.10)
80+	1.05 (0.92-1.20)	0.97 (0.85 – 1.11)
**eGFR category (ml/min/1.73m^2)**		
≥60	ref	ref
45-59	1.10 (0.99 – 1.23)	1.13 (1.01 – 1.27)
30-44	1.25 (1.10 – 1.42)	1.29 (1.14 - 1.46)
15-29	1.65 (1.33 – 2.06)	1.74 (1.40 - 2.16)
**Comorbidities**		
Hypertension	1.03 (0.93 – 1.13)	0.98 (0.88 – 1.09)
Ischaemic Heart Disease	1.00 (0.92 – 1.09)	0.98 (0.89 – 1.07)
Heart Failure	1.05 (0.96 – 1.15)	1.01 (0.92 – 1.11)
Arrhythmia	1.09 (1.00 – 1.19)	1.07 (0.98 – 1.18)
Diabetes	1.06 (0.97 – 1.16)	1.05 (0.95 – 1.15)
Peripheral Arterial Disease	1.12 (0.98 – 1.27)	1.08 (0.93 – 1.24)
**Calendar time**		
2004-2006	ref	ref
2007-2009	1.22 (1.07 – 1.38)	1.21 (1.07 – 1.38)
2010-2014	1.34 (1.17 – 1.53)	1.35 (1.18 – 1.55)
**Baseline potassium**		
<5.0mmol/L	ref	ref
5.0-5.5mmol/L	1.05 (0.91 – 1.20)	0.98 (0.86 – 1.12)
>5.5mmo/L	0.98 (0.73 – 1.30)	0.90 (0.68 -1.20)

eGFR – estimated Glomerular Filtration Rate.
Fully adjusted: adjusted for age, gender, CKD stage, hypertension, heart failure, ischaemic heart disease, arrhythmias, diabetes, peripheral arterial disease, baseline potassium and calendar time. Further adjustment for lifestyle covariates made marginal difference to all results, thus these variables are not included in models shown.

For peer review only

For peer review only - http://bmjopen.bmj.com/site/about/guidelines.xhtml

Adverse biochemical values

Of those with follow-up monitoring within two months of AA initiation (n=6,520), 2.0% had hyperkalaemia, 2.7% a creatinine ≥220µmol/L and 13.5% a 30% increase in creatinine from baseline, on their first blood test. **(Table 4)**. Of those with creatinine ≥220µmol/L, 29 people (16%) had creatinine ≥220µmol/L at baseline. Approximately half of patients with hyperkalaemia discontinued AA within 30 days of the blood test **(Table 4)**. However, less than one third of those with a >30% increase in creatinine discontinued AA, compared to 43% of those with a post-initiation creatinine ≥220µmol/L. Among non-hospitalised patients, the proportions with adverse laboratory values were lower while the proportion of people discontinuing the AA with each adverse biochemical value was similar **(Supplementary Table 4)**.

Table 4: Proportion with adverse biochemical values on testing within two months of AA initiation and number subsequently discontinuing aldosterone antagonist

	Hyperkalaemia (≥6mmol/L)‡	Creatinine ≥220µmol/L	≥30% increase in creatinine
Number with adverse biochemical values*	128/6373 (2.0%)	177/6520 (2.7%)	877/6520 (13.5%)
Number discontinuing AA§	68/128 (53.1%)	76/177 (42.9%)	251/877 (28.6%)

*Serum potassium and creatinine values on first blood test within two months of AA initiation.
‡Missing data for 147 people (2.3%) for first follow-up potassium value.
§Discontinuation defined as no further prescriptions of AA after blood test plus 30 days.
AA–aldosterone antagonist.

Factors associated with adverse biochemical values

In fully adjusted logistic regression models, baseline eGFR<60mls/min/1.73m^2 and potassium ≥5mmol/L were independently associated with hyperkalaemia **(Table 5)**. Women had lower odds than men for a post-initiation creatinine ≥220µmol/L, but had a higher odds of a 30% increase in creatinine. In sensitivity analyses of the non-hospitalised cohort, similar associations between age and sex and the occurrence of adverse biochemical values were observed **(Supplementary Table 5)**.

For peer review only - http://bmjopen.bmj.com/site/about/guidelines.xhtml

For peer review only

Table 5: Associations between patient characteristics and adverse biochemical values after aldosterone antagonist initiation

	OR (95% CI)		
	Hyperkalaemia[‡*] (≥6mmol/L)	Creatinine[*] ≥220µmol/L	≥30% increase in creatinine[*]
**Female**	1.04 (0.70 – 1.54)	0.34 (0.23 – 0.51)	1.47 (1.25 – 1.73)
**Age (years)**			
<50	0.15 (0.02 – 1.08)	0.96 (0.32 – 2.84)	0.34 (0.21 – 0.55)
50-59	0.29 (0.10 – 0.86)	0.80 (0.32 – 2.84)	0.45 (0.32 – 0.65)
60-64	0.60 (0.28 – 1.31)	1.17 (0.53 – 2.56)	0.69 (0.49 – 0.96)
65-69	0.83 (0.43 – 1.59)	0.36 (0.16 – 0.83)	0.64 (0.49 – 0.85)
70-75	*ref*	*ref*	*ref*
76-79	0.75 (0.43 – 1.33)	0.71 (0.42 – 1.20)	0.94 (0.75 – 1.18)
80+	0.69 (0.40 – 1.19)	0.50 (0.30 – 0.83)	1.10 (0.87 – 1.39)
**eGFR category (ml/min/1.73m^2)**			
≥60	*ref*	*ref*	*ref*
45-59	2.06 (1.26 – 3.35)	2.80 (1.22 – 6.39)	0.81 (0.66 – 0.99)
30-44	2.32 (1.31 – 4.11)	20.83 (9.73 – 44.58)	0.82 (0.67 – 1.00)
15-29	3.62 (1.60 – 8.22)	248.0 (117.2-524.7)	1.01 (0.72 – 1.40)
**Comorbidities**			
Hypertension	1.27 (0.76 – 2.14)	0.93 (0.61 – 1.41)	1.18 (0.98 – 1.41)
Ischaemic Heart Disease	0.67 (0.45 – 1.00)	0.60 (0.43 – 0.85)	0.95 (0.82 – 1.11)
Heart Failure	0.77 (0.53 – 1.14)	0.97 (0.67 – 1.41)	1.12 (0.96 – 1.31)
Arrhythmia	1.00 (0.68 – 1.46)	0.81 (0.57 – 1.15)	1.11 (0.96 – 1.28)
Diabetes	1.21 (0.83 – 1.76)	1.11 (0.80 – 1.55)	1.18 (1.01 – 1.38)
Peripheral Arterial Disease	0.82 (0.44-1.51)	1.04 (0.64- 1.68)	1.09 (0.85 – 1.40)
**Calendar time**			
2004-2006	*ref*	*ref*	*ref*
2007-2009	1.14 (0.65-2.01)	1.36 (0.84 - 2.22)	0.97 (0.80 – 1.18)
2010-2014	1.08 (0.64- 1.83)	0.93 (0.58 – 1.51)	0.95 (0.78 – 1.15)
**Baseline potassium**			
<5.0mmol/L	*ref*		
5.0-5.5mmol/L	3.59 (2.43 - 5.32)	*n/a*	*n/a*
>5.5mmo/L	3.29 (1.57 – 6.88)		

*Adverse serum potassium and creatinine values on first blood test within two months of AA initiation.
N = 6,520 except ‡missing data for 147 people for first follow-up potassium value.
Only fully adjusted model is shown for clarity. Adjusted for age, gender, CKD stage, hypertension, heart failure, ischaemic heart disease, arrhythmias, diabetes, peripheral arterial disease, baseline potassium (hyperkalaemia model only) and calendar time. Further adjustment for lifestyle covariates made marginal difference to all results and is not included.
eGFR–estimated Glomerular Filtration Rate.

For peer review only

For peer review only - http://bmjopen.bmj.com/site/about/guidelines.xhtml

DISCUSSION

In this cohort study of 10,546 users of ACEI/ARB, less than a third had follow up blood testing within two weeks of AA initiation. Less than two-thirds had blood testing within two months, and of these 2% developed severe hyperkalaemia and a similar proportion developed an absolute value of creatinine at which some guidelines recommend AA cessation is considered. In the same time frame, 13.5% of the cohort developed a ≥30% increase in serum creatinine from baseline.

Strengths and Weaknesses

We used a linked primary and secondary care dataset to ensure complete information on comorbid diagnoses and to allow sensitivity analyses for those without hospital admissions. These data had high levels of completeness for baseline renal function and comorbidities allowing the majority of ACEI/ARB users initiating an AA to be included in the cohort. A limitation of our study was that we could only examine adverse biochemical values for those who had a record of subsequent blood testing in primary care. The risk of adverse values may have been different in patients who were not tested, or tested in secondary care. Lack of access to blood test results from secondary care could have created a bias towards missing data among the sickest patients and an underestimation of the rate of adverse biochemical values. However, restricting the analysis to individuals without any record of a hospitalisation in the 30-day period prior to and after AA initiation did not substantially change our results. A further limitation is that for quantifying baseline renal function we could not include an indicator for Afro-Caribbean ethnicity in the eGFR calculation. However, we expect the impact of this to be small given that only 3% of the English population is of Afro-Caribbean ethnicity and the proportion is lower in the older age-group of this study.[23]

To be pragmatic we quantified testing for all patients against the NICE heart failure practical guidelines, although not all patients will have had heart failure as the indication for treatment. We chose not to limit to patients coded to have heart failure because comorbidity coding may not be complete, because coding may be related to routine care received[24], and also because there is a growing evidence base for use of AA in resistant hypertension.[2] In addition, even for those with coded heart failure we did not have details of patients echocardiographic findings so could not restrict to those with impaired left ventricular function, although this is the group in whom the benefits of treatment despite the risk of worsening of renal function are clearest.[25]

A strength of our study is that as well as examining the biochemical values of concern noted in current NICE practical guidelines for heart failure, we also assessed the incidence of a ≥30% increase in creatinine after initiation of AA. This outcome is clinically familiar from monitoring of ACEI/ARB initiation but is not discussed in the practical guidelines as a parameter for consideration of the

For peer review only - http://bmjopen.bmj.com/site/about/guidelines.xhtml

benefits of continuing AA therapy. The clinical significance of such deteriorations in eGFR in this context are not clear and may indicate favourable haemodynamic changes associated with improved outcomes [6]. However, worsening of renal function has been shown to have prognostic significance after initiation of ACEI/ARB treatment [26], particularly in the context of heart failure with preserved ejection fraction.[25]

Comparison with other studies

In a study of heart failure patients receiving health care through the Veterans Affairs health insurance programme in the US between 2003 and 2013, blood testing was carried out in 42% of patients within two weeks of initiation of AA.[10] Two other studies of heart failure patients carried out in North American settings found slightly higher monitoring rates for AA initiators; 53.5% within 7 days of initiation[3] and 44.6% within 10 days of initiation.[11] The contrast between our results and the results of North American research is noteworthy because it has been suggested in the past that monitoring in the UK is more frequent than in Canada.[4, 9] However, prior research on the frequency of monitoring in the UK was carried out in one geographical area and did not directly assess concordance with guideline recommended testing.[9]

Previous research examining patient characteristics associated with blood testing within 13 months of AA initiation found similar results to our study; that males, older patients and those with CKD had higher odds of receiving blood tests.[27] While diabetes was weakly associated with testing in our study (OR 1.2 in sensitivity analysis of non-hospitalised patients), the association was less pronounced than in prior research (OR 1.6).[27]

We found that 2% of those initiating an AA experienced hyperkalaemia (potassium ≥6mmol/L) on the first blood testing event within two months of AA initiation. This compares to a rate of 2% found in RALES and 5.5% in the Eplenerone Post–Acute Myocardial Infarction Heart Failure Efficacy and Survival Study (EPHESUS), both clinical trials of AAs in patients with heart failure.[1, 28] In observational studies, rates of hyperkalaemia ranging from 6% [29] to 10% [30] have been reported. Such populations are said to be more reflective of the typical user of an AA in clinical practice than in clinical trials, and thus, the rates are thought to be more realistic.[4] The disparity between our results for hyperkalaemia and those found in other observational research may have stemmed from unknown rates of adherence in this unselected population, from differences in doses used [6], or from differences in methodology. For example, we analysed potassium values only on the first blood test within two months whereas prior studies examined peak potassium values within three months of initiation [29], and peak values within the individuals’ entire treatment period.[30] As time since initiation increases it is increasingly likely that blood tests in primary care are performed

For peer review only

For peer review only - http://bmjopen.bmj.com/site/about/guidelines.xhtml

due to an intercurrent illness or other event that may itself have contributed to hyperkalaemia or reduced renal function. Thus, we opted to look at the first blood testing event within two months to examine as much as possible a causal association between the initiation of an AA and the adverse biochemical value. In line with previous literature, we found that baseline potassium value was a significant predictor of hyperkalaemia after initiation of AA [28, 29, 31], as was reduced renal function.[27, 28, 29]

As currently referenced in some guidelines (**Supplementary Table 1**), using a threshold creatinine value as a safety indicator for renal function may not be an optimal measure given that absolute creatinine levels create an age, gender and ethnicity bias. Importantly we found that, consistent with a lower muscle mass, women had a lower odds of developing a creatinine ≥220µmol/L post AA initiation in comparison to men, but a 1.5 fold higher odds of a ≥30% increase in creatinine. The clinical implications of higher odds of ≥30% increase in creatinine are unknown but it is important to establish that there is not a discrepancy in related clinical outcomes. However, gender related differences in adverse biochemical values was not a pre-specified hypothesis so this finding must be considered hypothesis-generating.

Conclusions and clinical implications

We found that among patients prescribed an ACEI/ARB, less than one third of those initiating an AA received follow-up blood tests within two weeks and less than two-thirds within two months. Approximately 2% of patients developed potassium ≥6mmol/L or creatinine ≥220µmol/L on the first blood test within two months but 13.5% experienced a ≥30% increase in creatinine from baseline. Women had almost 50% higher odds of a ≥30% increase in creatinine than men. Baseline potassium levels >5.0mmol/L and baseline eGFR <60mls/min/1.73m^2 were independently associated with a potassium value ≥6mmol/L after AA initiation. Our results highlight the need for better adherence to monitoring guidelines, particularly for those in these high risk groups, and the importance of understanding the prognostic implications of changes in renal function after AA initiation.

For peer review only - http://bmjopen.bmj.com/site/about/guidelines.xhtml

303 **DATA SHARING**

304 No additional data available

305

306 **FIGURE TITLE AND LEGEND**

307 **Figure 1: Flowchart demonstrating cohort selection**

308 ACEI/ARB–angiotensin converting enzyme inhibitor/angiotensin receptor blocker, HES–hospital episode statistics, CPRD –

309 Clinical Practice Research Datalink, AA-aldosterone antagonist, ESRD–end stage renal disease

310

311

For peer review only

For peer review only - http://bmjopen.bmj.com/site/about/guidelines.xhtml

REFERENCES

1 Pitt B, Zannad F, Remme WJ, et al. The Effect of Spironolactone on Morbidity and Mortality in Patients with Severe Heart Failure. *New England Journal of Medicine* 1999;**341**:709-17.

2 Williams B, MacDonald TM, Morant S, et al. Spironolactone versus placebo, bisoprolol, and doxazosin to determine the optimal treatment for drug-resistant hypertension (PATHWAY-2): a randomised, double-blind, crossover trial. *The Lancet* 2015;**386**:2059-68.

3 Allen LA, Shetterly S, Peterson PN, et al. Guideline concordance of testing for hyperkalemia and kidney dysfunction during initiation of mineralocorticoid receptor antagonist therapy in patients with heart failure. *Circulation: Heart Failure* 2013;**7**:43-50.

4 Juurlink DN, Mamdani MM, Lee DS, et al. Rates of hyperkalemia after publication of the Randomized Aldactone Evaluation Study. *New England Journal of Medicine* 2004;**351**:543-51.

5 MHRA. Drug Safety Notice. Spironolactone and renin angiotensin system drugs in heart failure: risk of potentially fatal hyperkalaemia. Available at https://www.gov.uk/drug-safety-update/spironolactone-and-renin-angiotensin-system-drugs-in-heart-failure-risk-of-potentially-fatal-hyperkalaemia 2016.

6 Pitt B, Rossignol P. The safety of mineralocorticoid receptor antagonists (MRAs) in patients with heart failure. *Expert opinion on drug safety* 2016;**15**:659-65.

7 National Institute for Health and Clinical Excellence (NICE). NICE Guideline CG108 Chronic Heart Failure. Appendix D – Practical Notes. Available at https://www.nice.org.uk/guidance/cg108/evidence/full-guideline-appendix-d-practical-notes-separate-file-136060532 2010.

8 National Institute for Health and Clinical Excellence (NICE). NICE Guideline CG127 Hypertension: Clinical management of primary hypertension in adults. 2011.

9 Wei L, Struthers AD, Fahey T, et al. Spironolactone use and renal toxicity: population based longitudinal analysis. *BMJ* 2010;**340**:c1768.

10 Chauhan V, Dev S, Pham M, et al. Facility variation and predictors of serum potassium monitoring after initiation of a mineralocorticoid receptor antagonist in patients with heart failure. *American heart journal* 2015;**170**:543-9.

11 Cooper LB, Hammill BG, Peterson ED, et al. Consistency of Laboratory Monitoring During Initiation of Mineralocorticoid Receptor Antagonist Therapy in Patients With Heart Failure. *JAMA* 2015;**314**:1973-5.

12 Herrett E, Gallagher AM, Bhaskaran K, et al. Data resource profile: clinical practice research datalink (CPRD). *International journal of epidemiology* 2015;**44**:827-36.

13 Herrett E, Thomas SL, Schoonen WM, et al. Validation and validity of diagnoses in the General Practice Research Database: a systematic review. *British journal of clinical pharmacology* 2010;**69**:4-14.

14 Herrett E, Shah AD, Boggon R, et al. Completeness and diagnostic validity of recording acute myocardial infarction events in primary care, hospital care, disease registry, and national mortality records: cohort study. *BMJ* 2013;**346**:f2350.

15 Schmidt M, Mansfield KE, Bhaskaran K, et al. Adherence to guidelines for creatinine and potassium monitoring and discontinuation following renin–angiotensin system blockade: a UK general practice-based cohort study. *BMJ Open* 2017;**7**:e012818.

16 McDonald H. The epidemiology of infections among older people with diabetes mellitus and chronic kidney disease. London School of Hygiene & Tropical Medicine 2015.

17 Bhaskaran K, Douglas I, Forbes H, et al. Body-mass index and risk of 22 specific cancers: a population-based cohort study of 5· 24 million UK adults. *The Lancet* 2014;**384**:755-65.

18 Levey AS, Stevens LA, Schmid CH, et al. A New Equation to Estimate Glomerular Filtration Rate. *Annals of internal medicine* 2009;**150**:604-12.

19 Clase CM, Barzilay J, Gao P, et al. Acute change in glomerular filtration rate with inhibition of the renin-angiotensin system does not predict subsequent renal and cardiovascular outcomes. *Kidney International* 2016.

For peer review only - http://bmjopen.bmj.com/site/about/guidelines.xhtml

20 National Institute for Health and Clinical Excellence (NICE). NICE guideline CG182 - Chronic kidney disease in adults: assessment and management. 2014.

21 Mathur R, Bhaskaran K, Chaturvedi N, et al. Completeness and usability of ethnicity data in UK-based primary care and hospital databases. *Journal of public health* 2014;**36**:684-92.

22 StataCorp. Stata Statistical Software: Release 14. College Station, TX: StataCorp LP. 2015.

23 Office for National Statistics. Census: Key Statistics for local authorities in England and Wales, table number KS201EW [online]. 2012. Available from: www.ons.gov.uk/Fons/Frel/Fcensus/F2011-census/Fkey-statistics-for-local-authorities-in-england-and-wales/Frft-table-ks201ew.xls. 2012.

24 Nitsch D CB, Hull S and Wheeler DC on behalf of the National CKD Audit and Quality Improvement Programme in Primary Care,. National Chronic Kidney Disease Audit - National Report (Part 1). Available from https://www.hqip.org.uk/public/cms/253/625/19/704/08532%20CKD%20Audit%20Report%20Jan%2017%20FINAL.pdf?realName=HtoEm0.pdf&v=0. 2017.

25 Beldhuis IE, Streng KW, Ter Maaten JM, et al. Renin-Angiotensin System Inhibition, Worsening Renal Function, and Outcome in Heart Failure Patients With Reduced and Preserved Ejection Fraction: A Meta-Analysis of Published Study Data. *Circulation Heart failure* 2017;**10**.

26 Schmidt M, Mansfield KE, Bhaskaran K, et al. Serum creatinine elevation after renin-angiotensin system blockade and long term cardiorenal risks: cohort study. *BMJ* 2017;**356**:j791.

27 Raebel MA, McClure DL, Chan KA, et al. Laboratory evaluation of potassium and creatinine among ambulatory patients prescribed spironolactone: are we monitoring for hyperkalemia? *Annals of Pharmacotherapy* 2007;**41**:193-200.

28 Pitt B, Bakris G, Ruilope LM, et al. Serum Potassium and Clinical Outcomes in the Eplerenone Post–Acute Myocardial Infarction Heart Failure Efficacy and Survival Study (EPHESUS). *Circulation* 2008;**118**:1643-50.

29 Shah KB, Rao K, Sawyer R, et al. The adequacy of laboratory monitoring in patients treated with spironolactone for congestive heart failure. *Journal of the American College of Cardiology* 2005;**46**:845-9.

30 Svensson M, Gustafsson F, Galatius S, et al. How prevalent is hyperkalemia and renal dysfunction during treatment with spironolactone in patients with congestive heart failure? *Journal of cardiac failure* 2004;**10**:297-303.

31 Tamirisa KP, Aaronson KD, Koelling TM. Spironolactone-induced renal insufficiency and hyperkalemia in patients with heart failure. *American heart journal* 2004;**148**:971-8.

For peer review only

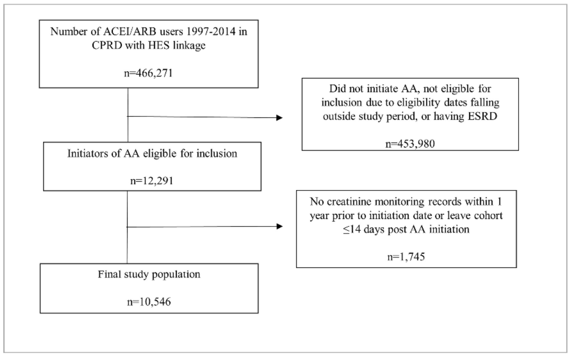

Title Figure 1: Flowchart demonstrating cohort selection
Legend ACEI/ARB–angiotensin converting enzyme inhibitor/angiotensin receptor blocker, HES–hospital episode statistics, CPRD – Clinical Practice Research Datalink, AA-aldosterone antagonist, ESRD–end stage renal disease

338x190mm (300 x 300 DPI)

For peer review only

Supplementary Information

Table S1: Summary of clinical guideline recommendations for blood testing after initiation of AA

	NICE HF*	NICE HF Practical Guidelines Appendix D	NICE HF Practical Guidelines Appendix M	NICE Hypertension§	British National Formulary	American Guidelines∞	European Guidelines
≤7 days	*na*	x	x	*na*	x	x	x
≤1 month	*na*	x	x	x	X	x	x
≤2 month	*na*	x	x	*na*	X	x	x
≤3 month	*na*	x	x	*na*	X	x	x
≤6 month	*na*	x	x	*na*	X	x	x
≤9 month	*na*	*na*	x	*na*	X	x	x
≤12 month	*na*	x	x	*na*	X	x	x
How frequently after 1st year	*na*	6 monthly	6 monthly	*na*	6 monthly	3 monthly	4 monthly
Discontinue if K≥6mmol/L	*na*	x	x	*na*	*na*	x∞∞	x
Discontinue if creatinine ≥220µmol/L	*na*	x	x†	*na*	*na*	*na***	x‡
Discontinue if relative changes between baseline creatinine and follow up creatinine or eGFR ≥X%	*na*	*na*	*na*	*na*	*na*	*na***	*na*

* NICE HF guidelines recommend monitoring within two weeks after a medication change, but refer to Appendix D for guidance on monitoring for hyperkalaemia and renal function deterioration

† Recommends discontinuing if creatinine ≥200µmol/L

§ Recommends blood testing as required after testing within one month of AA initiation/increased diuretic use

∞ Recommends blood testing within 2-3 days of AA initiation, and again at 7 days.

∞∞ Recommends discontinuation at potassium > 5.5mEq/L

** Does not provide strict stopping/starting criteria according to serum creatinine thresholds. Suggests that risk of hyperkalaemia increases with worsening renal function and in elderly patients, or patients with low muscle mass in whom serum creatinine does not accurately reflect GFR, determination that GFR is >30ml/min/1.73m^2 is recommended.

For peer review only - http://bmjopen.bmj.com/site/about/guidelines.xhtml

‡ Recommends discontinuing if creatinine ≥310µmol/L or eGFR <20ml/min/1.73m^2
European Guidelines: "2016 ESC Guidelines for the diagnosis and treatment of acute and chronic heart failure"

American Guidelines: "2013 ACCF/AHA Guideline for the Management of Heart Failure"
NICE: National Institute for Health and Care Excellence
HF: Heart Failure
GP: General Practitioner
"x" refers to when the row condition is satisfied, "na" refers to not mentioned or not satisfied.

For peer review only

SI Table 2: Proportion of non-hospitalised patients taking ACEI/ARB who initiate aldosterone antagonists in UK primary care 2004-2014 with blood tests with 14 days of AA initiation

Non-hospitalised population	Monitoring ≤14 days post initiation (n., %)	No monitoring ≤14 days post initiation (n., %)
n=5787	1892 (32.7)	3897 (67.3)

Proportions for monitoring use 5787 as denominator. Non-hospitalisation refers to not having a hospitalisation in the 30 days prior to or post initiation of AA.

For peer review only

For peer review only - http://bmjopen.bmj.com/site/about/guidelines.xhtml

SI Table 3: Association between non-hospitalised patient characteristics and follow up monitoring within two weeks of initiation of an aldosterone antagonist

	OR (95% CI)	
	**Age and sex adjusted**	**Fully adjusted**
**Male**	*ref*	*ref*
**Female**	0.88 (0.78 – 0.99)	0.91 (0.80 – 1.03)
**Age (years)**		
<50	0.38 (0.28 – 0.53)	0.42 (0.30 – 0.58)
50-59	0.69 (0.56 – 0.85)	0.75 (0.60 – 0.93)
60-64	0.83 (0.66 – 1.06)	0.89 (0.70 – 1.13)
65-69	0.93 (0.76 – 1.14)	0.95 (0.77 – 1.17)
70-75	*ref*	*ref*
76-79	0.91 (0.75 – 1.11)	0.92 (0.75 – 1.12)
80+	1.13 (0.95 – 1.34)	1.11 (0.92 – 1.32)
**eGFR category (ml/min/1.73m^2)**		
≥60	*ref*	*ref*
45-59	1.00 (0.86 – 1.16)	1.02 (0.87 – 1.19)
30-44	1.10 (0.93 – 1.31)	1.10 (0.92 – 1.32)
15-29	1.13 (0.82 – 1.57)	1.15 (0.83 – 1.60)
**Comorbidities**		
Hypertension	0.98 (0.86 – 1.12)	0.96 (0.83 – 1.60)
Ischaemic Heart Disease	1.09 (0.97 – 1.23)	1.04 (0.93 – 1.17)
Heart Failure	1.17 (1.04 – 1.32)	1.11 (0.97 – 1.26)
Arrhythmia	1.16 (1.03 – 1.31)	1.10 (0.97 – 1.25)
Diabetes	1.16 (1.02 – 1.33)	1.17 (1.02 – 1.34)
Peripheral Arterial Disease	1.13 (0.93 – 1.38)	1.06 (0.87 – 1.31)
**Calendar time**		
2004-2006	*ref*	*ref*
2007-2009	1.26 (1.07 – 1.49)	1.25 (1.06 – 1.49)
2010-2014	1.36 (1.15 – 1.61)	1.35 (1.13 – 1.60)
**Baseline potassium**		
<5.0mmol/L	*ref*	*ref*
5.0-5.5mmol/L	1.05 (0.87 – 1.27)	1.01 (0.84 – 1.23)
>5.5mmo/L	0.87 (0.59 – 1.30)	0.85 (0.57 – 1.26)

eGFR–estimated Glomerular Filtration Rate.
Fully adjusted: adjusted for age, gender, CKD stage, hypertension, heart failure, ischaemic heart disease, arrhythmias, diabetes, peripheral arterial disease, baseline potassium and calendar time. Further adjustment for lifestyle covariates and socioeconomic status made marginal difference to all results, thus these variables are not included in models shown.

For peer review only - http://bmjopen.bmj.com/site/about/guidelines.xhtml

SI Table 4: Proportion of non-hospitalised patients with adverse biochemical values on testing within 2 months of AA initiation and number subsequently discontinuing aldosterone antagonist

	Hyperkalaemia (>=6mmol/L)‡	Creatinine (≥220µmol/L)	≥30% Change in Creatinine
Number with adverse biochemical values (n,%)*	60/3698 (1.6%)	75/3757 (2.0%)	374/3757 (10.0%)
Number discontinuing AA (n, %)∞	30/60 (50.0%)	29/75 (38.7%)	109/374 (29.1%)

*serum potassium and creatinine values on first blood test within two months of AA initiation.
‡Missing data for 59 people for first follow-up potassium value.
§Discontinuation defined as no further prescriptions of AA after blood test plus 30 days.
AA–aldosterone antagonist.

For peer review only

For peer review only - http://bmjopen.bmj.com/site/about/guidelines.xhtml

SI Table 5: Associations between non-hospitalised patient characteristics and adverse biochemical values after aldosterone antagonist initiation

	OR (95% CI)		
	**Hyperkalaemia* (>=6mmol/L)**	**Creatinine* (≥220μmol/L)**	**≥30% Change in Creatinine**
**Male**	*ref*	*ref*	*ref*
**Female**	1.53 (0.93 – 2.51)	0.78 (0.50 – 1.23)	1.52 (1.22 – 1.90)
**Age (years)**			
<50	-	0.80 (0.22 – 2.87)	0.37 (0.16 – 0.85)
50-59	0.47 (0.15 – 1.45)	0.45 (0.16 – 1.30)	0.67 (0.40 – 1.13)
60-64	0.57 (0.18 – 1.79)	0.67 (0.23 – 1.94)	0.79 (0.47 – 1.30)
65-69	0.41 (0.13 – 1.28)	0.20 (0.05 – 0.81)	0.81 (0.52 – 1.26)
70-75	*ref*	*ref*	*ref*
76-79	0.93 (0.44 - 1.97)	1.34 (0.65 – 2.78)	1.05 (0.73 – 1.51)
80+	0.82 (0.41 - 1.64)	1.22 (0.63 – 2.37)	1.25 (0.87 – 1.80)
**eGFR category (ml/min/1.73m^2)**			
≥60	*ref*		
45-59	3.36 (1.40 – 8.05)	5.02 (1.24 – 20.24)	0.84 (0.64 – 1.10)
30-44	6.01 (2.42 – 14.89)	47.79 (13.70 – 166.76)	1.11 (0.81 – 1.53)
15-29	8.69 (2.51 – 30.10)	875.60 (240.46 – 3188.32)	1.23 (0.75 – 2.04)
**Comorbidities**			
Hypertension	1.11 (0.51 – 2.37)	1.12 (0.60 -2.08)	1.30 (0.98 - 1.73)
Ischaemic Heart Disease	0.52 (0.28 – 0.96)	0.59 (0.33 – 1.07)	1.03 (0.84 -1.27)
Heart Failure	0.72 (0.42 – 1.22)	0.98 (0.55 -1.73)	0.92 (0.73 - 1.15)
Arrhythmia	0.93 (0.54 – 1.61)	0.57 (032 – 1.01)	1.03 (0.82 -1.29)
Diabetes	0.96 (0.53 – 1.74)	1.10 (0.61 – 1.98)	0.97 (0.76 – 1.24)
Peripheral Arterial Disease	1.05 (0.38 – 2.87)	0.55 (0.21 – 1.40)	0.89 (0.59 - 1.34)
**Calendar time**			
2004-2006			
2007-2009	0.94 (0.42 – 2.07)	1.19 (0.60 – 2.38)	1.01 (0.75 – 1.35)
2010-2014	0.91 (0.46 – 1.77)	0.76 (0.40 – 1.47)	0.96 (0.72 – 1.27)
**Baseline potassium**			
<5.0mmol/L			
5.0-5.5mmol/L	2.52 (1.31 – 4.83)	*n/a*	*n/a*
>5.5mmo/L	5.26 (1.87 – 14.81)		

*Adverse serum potassium and creatinine values on first blood test within two months of AA initiation.
N = 3,757 except missing data for 59 people for first follow-up potassium value.
Only fully adjusted model is shown for clarity. Adjusted for age, gender, CKD stage, hypertension, heart failure, ischaemic heart disease, arrhythmias, diabetes, peripheral arterial disease, baseline potassium (hyperkalemia model only) and calendar time. Further adjustment for lifestyle covariates and socioeconomic status made marginal difference to all results and is not presented.
eGFR–estimated Glomerular Filtration Rate.

For peer review only - http://bmjopen.bmj.com/site/about/guidelines.xhtml

STROBE Statement—checklist of items that should be included in reports of observational studies

	Item No	Recommendation	Page
**Title and abstract**	1	(*a*) Indicate the study's design with a commonly used term in the title or the abstract	1
		(*b*) Provide in the abstract an informative and balanced summary of what was done and what was found	3
**Introduction**			
Background/rationale	2	Explain the scientific background and rationale for the investigation being reported	5
Objectives	3	State specific objectives, including any prespecified hypotheses	5
**Methods**			
Study design	4	Present key elements of study design early in the paper	6
Setting	5	Describe the setting, locations, and relevant dates, including periods of recruitment, exposure, follow-up, and data collection	6+7
Participants	6	(*a*) *Cohort study*—Give the eligibility criteria, and the sources and methods of selection of participants. Describe methods of follow-up<br>*Case-control study*—Give the eligibility criteria, and the sources and methods of case ascertainment and control selection. Give the rationale for the choice of cases and controls<br>*Cross-sectional study*—Give the eligibility criteria, and the sources and methods of selection of participants	6
		(*b*) *Cohort study*—For matched studies, give matching criteria and number of exposed and unexposed<br>*Case-control study*—For matched studies, give matching criteria and the number of controls per case	na
Variables	7	Clearly define all outcomes, exposures, predictors, potential confounders, and effect modifiers. Give diagnostic criteria, if applicable	7+6
Data sources/ measurement	8*	For each variable of interest, give sources of data and details of methods of assessment (measurement). Describe comparability of assessment methods if there is more than one group	6
Bias	9	Describe any efforts to address potential sources of bias	8
Study size	10	Explain how the study size was arrived at	10 (flowchart)
Quantitative variables	11	Explain how quantitative variables were handled in the analyses. If applicable, describe which groupings were chosen and why	6
Statistical methods	12	(*a*) Describe all statistical methods, including those used to control for confounding	8
		(*b*) Describe any methods used to examine subgroups and interactions	8
		(*c*) Explain how missing data were addressed	6
		(*d*) *Cohort study*—If applicable, explain how loss to follow-up was addressed<br>*Case-control study*—If applicable, explain how matching of cases and controls was addressed<br>*Cross-sectional study*—If applicable, describe analytical methods	

For peer review only

taking account of sampling strategy	
(*e*) Describe any sensitivity analyses	8

**Results**			**Page**
Participants	13*	(a) Report numbers of individuals at each stage of study—eg numbers potentially eligible, examined for eligibility, confirmed eligible, included in the study, completing follow-up, and analysed	Flowchart pg 10
		(b) Give reasons for non-participation at each stage	Flowchart pg 10
		(c) Consider use of a flow diagram	Flowchart pg 10
Descriptive data	14*	(a) Give characteristics of study participants (eg demographic, clinical, social) and information on exposures and potential confounders	Table1
		(b) Indicate number of participants with missing data for each variable of interest	Table 1 pg 11
		(c) *Cohort study*—Summarise follow-up time (eg, average and total amount)	Na
Outcome data	15*	*Cohort study*—Report numbers of outcome events or summary measures over time	Table 2+4
		Case-control study—Report numbers in each exposure category, or summary measures of exposure	
		Cross-sectional study—Report numbers of outcome events or summary measures	
Main results	16	(*a*) Give unadjusted estimates and, if applicable, confounder-adjusted estimates and their precision (eg, 95% confidence interval). Make clear which confounders were adjusted for and why they were included	Tables 3 +5
		(*b*) Report category boundaries when continuous variables were categorized	Tables 3+5
		(*c*) If relevant, consider translating estimates of relative risk into absolute risk for a meaningful time period	Na
Other analyses	17	Report other analyses done—eg analyses of subgroups and interactions, and sensitivity analyses	12+15
**Discussion**			
Key results	18	Summarise key results with reference to study objectives	16
Limitations	19	Discuss limitations of the study, taking into account sources of potential bias or imprecision. Discuss both direction and magnitude of any potential bias	16
Interpretation	20	Give a cautious overall interpretation of results considering objectives, limitations, multiplicity of analyses, results from similar studies, and other relevant evidence	16-18
Generalisability	21	Discuss the generalisability (external validity) of the study results	16
**Other information**			
Funding	22	Give the source of funding and the role of the funders for the present study and, if applicable, for the original study on which the present article is based	2

*Give information separately for cases and controls in case-control studies and, if applicable, for exposed and unexposed groups in cohort and cross-sectional studies.

Note: An Explanation and Elaboration article discusses each checklist item and gives methodological background and published examples of transparent reporting. The STROBE checklist is best used in conjunction with this article (freely available on the Web sites of PLoS Medicine at http://www.plosmedicine.org/, Annals of Internal Medicine at

For peer review only - http://bmjopen.bmj.com/site/about/guidelines.xhtml

http://www.annals.org/, and Epidemiology at http://www.epidem.com/). Information on the STROBE Initiative is available at www.strobe-statement.org.

For peer review only

For peer review only - http://bmjopen.bmj.com/site/about/guidelines.xhtml
